# Ethnopharmacology and therapeutic potential of *Anchusa strigosa*: a comprehensive review

**DOI:** 10.3389/fphar.2023.1301154

**Published:** 2023-11-24

**Authors:** Ziad Chebaro, Adnan Badran, Marc Maresca, Elias Baydoun, Joelle Edward Mesmar

**Affiliations:** ^1^ Department of Biology, American University of Beirut, Beirut, Lebanon; ^2^ Department of Nutrition, University of Petra, Amman, Jordan; ^3^ CNRS, Centrale Marseille, iSm2, Aix-Marseille University, Marseille, France

**Keywords:** *Anchusa strigosa*, traditional uses, herbal medicine, phytochemistry, pharmacology

## Abstract

*Anchusa strigosa* Banks and Sol. is a rough flowering plant of the Boraginaceae family native to Eastern Mediterranean region that is widely used in traditional herbal medicine, mainly for the treatment of wounds, abdominal pain, and arthritis, to name a few. This article aims to gather knowledge related to the medicinal properties of *A. strigosa*. Specifically, it summarizes its traditional uses and pharmacological activities in the treatment of various diseases. Moreover, its botanical, ecological, and phytochemical characteristics are also discussed. Research showed that this plant is rich in pyrrolizidine alkaloids, particularly in the leaves. Other bioactive metabolites identified in this plant include flavonoids, phenolic acids, triterpenes, organic acids, and volatile organic compounds. These phytochemicals are responsible for the reported pharmacological properties of *A. strigosa*, including antimicrobial, antioxidant, anticancer, anti-inflammatory, antiarthritic, gastric protective, antidiabetic, and pro-wound healing. This warrants further investigation into the molecular mechanism of action behind the observed effects to elucidate its therapeutic potential. Nevertheless, more research on this plant is needed to ensure its efficacy and safety.

## 1 Introduction

Historically, plants and natural products have been widely used in folk medicine as traditional remedies. Moreover, their use in primary healthcare has been expanding rapidly, with complementary and alternative medicine becoming mainstream in both developing and developed countries due to the wide acceptance of natural remedies and their perception as generally safe. Now, plant-based natural products play an important role in modern drug development, owing to the diversity and structural complexity of their metabolites and their unique properties. In fact, plants have contributed to the development of many drugs either directly or indirectly by using the core structure of natural bioactive metabolites as scaffolds. For example, morphine, which is found in *Papaver somniferum*, was the first natural product introduced and used as a therapeutic drug in 1826. And aspirin was developed as a semi-synthetic drug in 1899 to treat pain, fever, and inflammation, as a derivative of salicilin from *Salix alba*. Other examples of plant-derived drugs include Paclitaxel from *Taxus brevifolia*, which is chemotherapeutic agent used for the treatment of various cancers, and Artemisinin from *Artemisia annua*, used for the treatment of multidrug-resistant malaria, to name a few.

*Anchusa* is a major genus of rough flowering plants that belongs to the Boraginaceae family, with around 34 accepted species growing mainly in a temperate biome. It is native to Europe, the Middle East and North Africa region, Western Asia, and South Africa (Inflammation, 2023). Species of this genus have been used in traditional medicine for the treatment of various ailments (Al-Snafi, 2014), including arthritis (Lev and Amar, 2000; El Beyrouthy et al., 2008; Polat et al., 2013), abdominal pain (Al-Khalil, 1995; Polat et al., 2013; Mükemre et al., 2015), kidney stones (Mordi et al., 2021), and for wound healing (Honda et al., 1996; Lardos, 2006; Hudaib et al., 2008; Qasem, 2015).

*A. officinalis* L., *A. strigosa* Banks and Sol., and *A. azurea* Mill. are the most studied species of this genus, with insight given into their ethnobotanical uses and their phytochemical and pharmacological properties. Of particular interest to this review is *A. strigosa*, where we provide an up-to-date and comprehensive overview of its phytochemical properties of *A. strigosa* and pharmacological activities, with the aim to expose its potential as an attractive source of medicinal agents and call for further investigation into its therapeutic value.

## 2 Methods

Literature search was conducted using the keywords and MeSH terms ‘Anchusa strigosa Banks & Sol.’2023, “A. strigosa,” AND (“phytochemical content,” “pharmacological properties, or activities, or effects, or roles,” “anti-inflammatory,” “antioxidant,” “anticancer,” “ethnopharmacology,” “traditional uses,” “medicinal uses,” “antimicrobial,” “antibacterial,” or “antifungal”) in major scientific literature databases such as PubMed, Scopus, ScienceDirect, SciFinder, Medicinal and Aromatic Plants Abstracts, Dr. Duke’s Phytochemical and Ethnobotanical Databases, Chemical Abstracts, and Henriette′s Herbal Homepage. Google and Google Scholar were also used for general web searches. The search period covered articles published between 1984 and 2023. The search yielded 23 research articles on *A. strigosa*.

## 3 Ehtnopharmacological uses

*A. strigosa* Banks & Sol. is a non-succulent and short-lived perennial plant widespread in the Eastern Mediterranean region, particularly found in Greece, Turkey, Israel, Lebanon, Syria, and Iran. It is commonly known as prickly alkanet or strigose bugloss, and as “lisan al-thawr,” “balghasoun,” or “himhim” in Arabic. It is a drought-hardy wild plant considered as a “famine food” and known for its traditional culinary applications, particularly in Palestinian cuisine (Qasem, 2015; Yeşil et al., 2019; Fullilove, 2022; Baydoun et al., 2023). It is additionally used in folk medicine practices (Yeşil et al., 2019; Baydoun et al., 2023) for the treatment of skin diseases (Said et al., 2002; Abu-Rabia, 2015), wounds (Dafni et al., 1984; Palevich, D and Yaniv, Z, 1991; Ali-Shtayeh et al., 1998; Said et al., 2002; Hudaib et al., 2008), arthritis (Ali-Shtayeh et al., 2000; Said et al., 2002; El Beyrouthy et al., 2008), and abdominal pain (Palevich, D and Yaniv, Z, 1991; Al-Khalil, 1995; Ali-Shtayeh et al., 1998; Abu-Rabia, 2015), among others. A summary of the traditional uses of *A. strigosa* is shown in Table 1.

**TABLE 1:** Ethnopharmacology of *Anchusa strigosa*.

Plant part	Mode of preparation	Traditional use	References
Leaves	Internal uptake of a standard decoction	- Used as a diuretic and analgesic	Said et al. (2002), Yesil and Inal (2021)
		- Treatment of respiratory infections and fever	
	External application of the juice from macerated leaves - Cataplasm of leaves	- Treatment of arthritis, skin diseases, wounds, irritation, and bone fracture	Dafni et al. (1984), Al-Khalil (1995), Said et al. (2002), El Beyrouthy et al. (2008)
Flowers	Internal uptake of a flower decoction	- Used as a diuretic, analgesic, diaphoretic, and sedative	Al-douri (2000)
Aerial parts	Internal uptake of a standard decoction	- Used as a anthelmintic	Hudaib et al. (2008)
		- Treatment of headaches	
	External application of bandages	- Treatment of wounds	Hudaib et al. (2008)
	Vapor	- Treatment of female sterility	Hudaib et al. (2008)
Roots	Internal uptake of a standard decoction	- Used as a diuretic, diaphoretic, and tonic	Palevich, D and Yaniv, Z (1991), Al-Khalil (1995), Ali-Shtayeh et al. (1998), Abu-Rabia (2015)
		- Treatment of abdominal pain and fever	
	External application of the juice from macerated crushed roots	- Used as a demulcent, antiseptic, and emollient	Palevich, D and Yaniv, Z (1991), Ali-Shtayeh et al. (1998), Abu-Rabia (2015)
		- Treatment of skin diseases, wounds, headaches, rheumatism, and edema	
Not specified	Inhalation of the tincture	- Treatment of chickenpox/varicella	Abu-Rabia (2015)
	water infusion and administering drops of the liquid as nasal drops	- Treatment of vomiting	Abu-Rabia (2015)

## 4 General characteristics

### 4.1 Botanical characteristics

**FIGURE 1:**
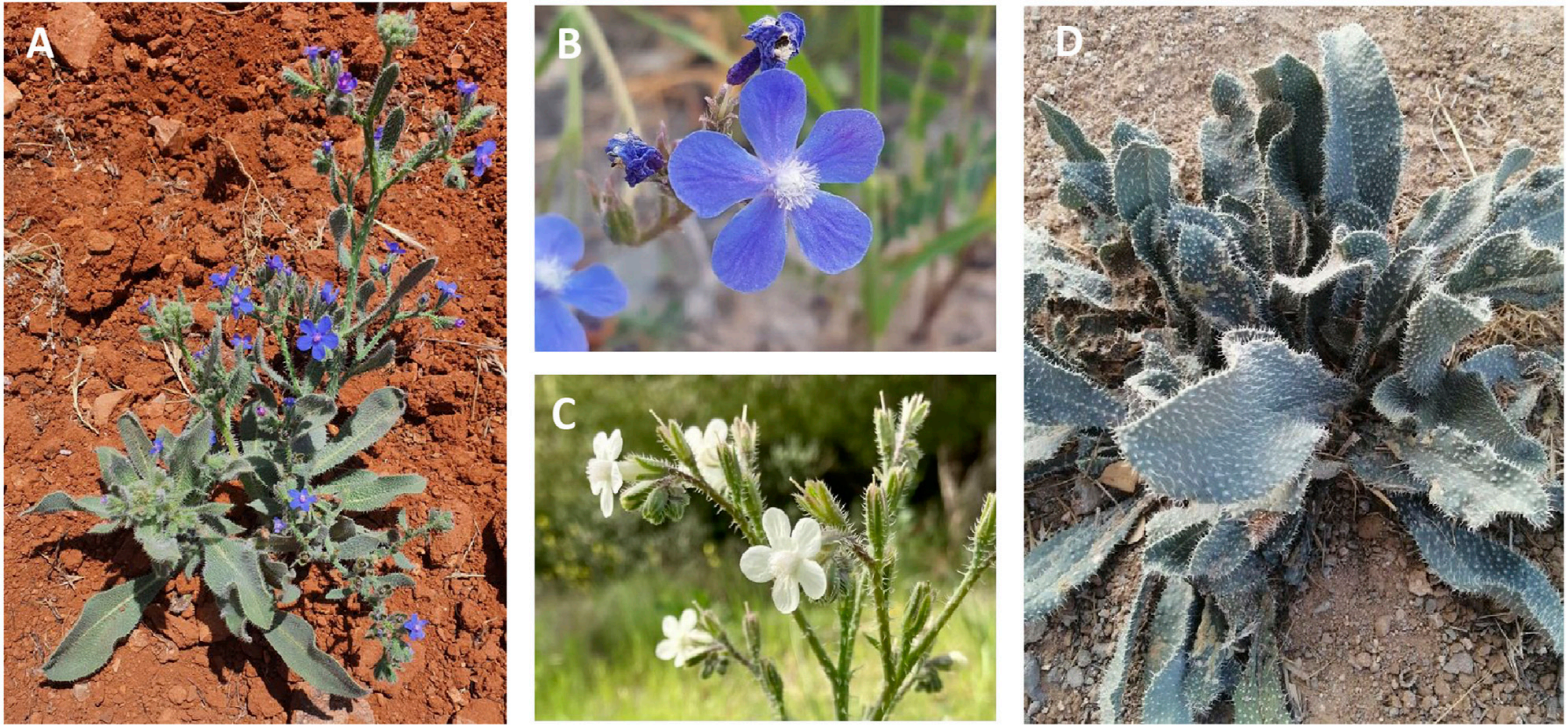
**(A)** *Anchusa strigosa* plant, **(B)** and **(C)** *Anchusa strigosa* flowers, and **(D)** *Anchusa strigosa* leaves. Images were obtained from https://www.gbif.org/occurrence/gallery?taxon_key=8192155 (accessed on 14 September 2023).

The taxonomic classification of *A. strigosa* is illustrated in Table 2. It is a hardy perennial weed with a bristly inflorescence stem that can grow up to a meter in height. It blooms in the late spring season from March to August, with trumpet-shaped flowers that are small (10–15 mm), tubular and distributed in an irregular pattern, but mostly dense clusters at the tips of the stems (Figure 1) (Tohmé and Tohmé, 2007). They can have a pale blue, violet, or white color, depending on the habitat. The age of the flower can also affect the color of the corolla, as it was observed that young flowers were violet, while mature ones were blue and produced a larger amount of nectar (Kadmon et al., 1991). The base of *A. strigosa* consists of a rosette of leaves that resemble the tongue of a ruminate by having a rough and a prickly texture, hence its Arabic designation “lisan al-thawr.” Moreover, it is characterized by oblong petiolate basal leaves and linear upper leaves, both covered in short and stiff hairs, as well as verrucose, exhibiting wart-like outgrowths. Finally, the roots of *A. strigosa* contain anchusin, a red-coloring dye that is used as food coloring and in cosmetics (Majumdar, D. N. and Chakravarty, G. C., 1940).

**TABLE 2:** Taxonomic classification of *Anchusa strigosa*.

Kingdom	Plantae
Phylum	Tracheophyta
Class	Magnoliopsida
Order	Boraginales
Family	Boraginaceae
Genus	*A* *n* *chusa* L.
Species	*A* *n* *chusa strigosa*
Binomial name	*A* *n* *chusa strigosa* Banks & Sol.

### 4.2 Ecological characteristics

The Mediterranean region harbors one of the richest sources of medicinal plants and hence has a long history of herbal remedies as an important source of treatment for several diseases (Saad et al., 2005). Of particular interest, *A. strigosa*, which is found in the Eastern Mediterranean region, naturally occurs in forests, rocky slopes, steppe vegetation, deserts, and along roadsides (Tohmé and Tohmé, 2007). It is native to Lebanon (Tohmé and Tohmé, 2007; El Beyrouthy et al., 2008; Baydoun et al., 2023; Chebaro et al., 2023), Syria (Alallan et al., 2018; Sulaiman et al., 2022), Palestine (Israel) (Dafni et al., 1984; Ali-Shtayeh et al., 1998, 2000; Lev and Amar, 2000; Said et al., 2002; Abutbul et al., 2005; Fullilove, 2022; Yarmolinsky et al., 2022), Jordan (Al-Khalil, 1995; Alali et al., 2007; Hudaib et al., 2008; Abbas et al., 2009; Dibas et al., 2017; Qasem, 2020; Al-Khatib et al., 2021), Iraq (Al-douri, 2000; Ghalib and Kadhim, 2021; Khit et al., 2023), Iran (Keshavarzi et al., 2017; Mordi et al., 2021), Turkey (Honda et al., 1996; Polat et al., 2013; Mükemre et al., 2015; Yeşil et al., 2019; Yesil and Inal, 2021), Greece (Selvi and Bigazzi, 2003), and Cyprus (Inflammation, 2023).

*A. strigosa* is a tenacious wild plant, with the ability to withstand periods of drought, and to adapt to a wide range of habitats, which is a characteristic of most plants of the Boraginaceae family. Due to its prominence in Palestinian cuisine and its ability to grow without human help and adapt to a wide range of habitats and conditions, such as disturbed soils and waste grounds, *A. strigosa* has been classified as a “famine food” (Fullilove, 2022). In fact, it is commonly used as food ingredient in conflict areas (Sulaiman et al., 2022), suggesting its important economic value and relevance in society.

### 4.3 Phytochemical characteristics

#### 4.3.1 Phytochemical composition

*A. strigosa* is a rich source of secondary metabolites, particularly pyrrolizidine alkaloids (PAs), which are a class of plant toxin found in wide variety of plant families, and notably among the Boraginaceae (Ibanez, 2005; Tamariz et al., 2018; Günthardt et al., 2021). They are essentially stored in plants as protoxins and play in important role in the plant defense response against insect herbivores and pathogens. In fact, in a study investigating the concentration of PAs in *A. strigosa*, it was shown that the leaves were the richest in PAs (23.63 mg/g of dried part), followed by the flowers (19.77 mg/g), and the roots (1.80 mg/g), further supporting the protective effects of PAs against pests (Siciliano et al., 2005). A summary of the PAs identified in *A. strigosa* is summarized in Table 3. PAs exhibit great structural variety, and their toxicity is mostly associated with the presence of a double bond in the necine base. These are referred to 1,2-unsaturated PAs and are metabolized to PA radicals in the intestine and liver, causing liver injury, mainly resulting in hepatic sinusoidal obstruction syndrome, as well as liver fibrosis (Neuman et al., 2015; Yang et al., 2019b, Yang et al., 2019a). They have also been linked to genotoxicity, neurological damage and potential tumorigenic effects in humans and animals (Chen et al., 2010). However, the beneficial use of PAs to treat diseases has been gaining interest in recent decades due to their numerous biological and pharmacological activities (Wei et al., 2021). For instance, PAs isolated from plants have been shown to possess anti-microbial, anti-inflammatory, anti-diabetic, anti-ulcer and anti-cancer properties, among others (Schramm et al., 2019). For example, indicine N-oxide has been used in the treatment of leukemia and PAs isolated from various plants have been shown to induce autophagy and apoptosis in several cancer cell lines including lung cancer cells and human colorectal cancer cells (Wei et al., 2021). Overall, PAs have indeed beneficial pharmacological properties with promising therapeutic applications. However, the use of PA-containing medicine is associated with many risks and should be used with caution. Their recommended dosage is still under debate and often lacks clinical evidence. Nevertheless, their potential use in cancer treatment has stimulated the interest of research groups to develop methods for the targeted delivery of PAs to cancer cells, therefore limiting their toxicity to the liver (drugtargetreview, 2022).

**TABLE 3:** Phytochemical composition of *Anchusa strigosa* extracts and their major metabolites.

Extract type	Analytical methods	Main results	Major metabolites	References
Whole plant				
Methanolic extract	HPLC, FTIR, TLC, and melting point	Identification of phenolic acids, flavonoids, and a pyrrolizidine alkaloid	Phenols:	Ghalib and. Kadhim (2021)
			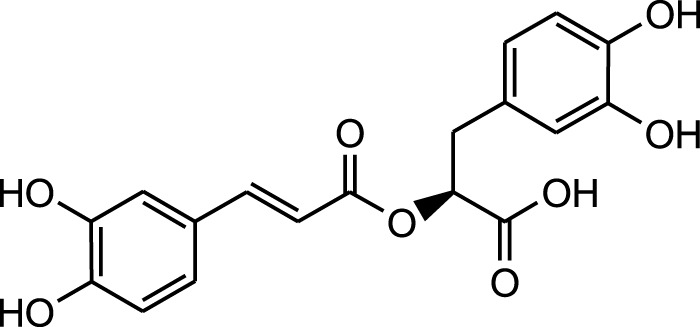 Rosmarinic acid	
			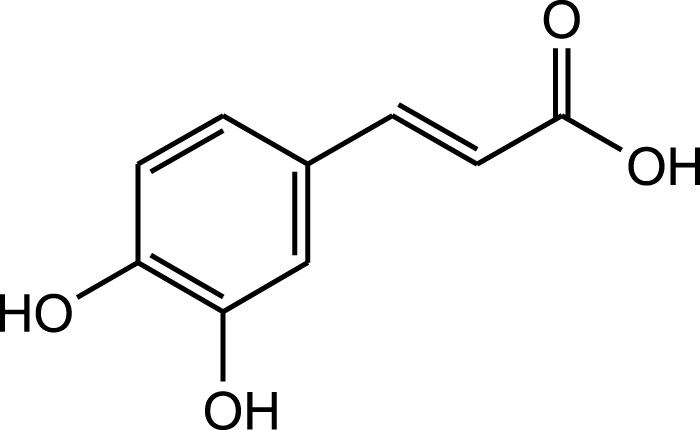	
			Caffeic acid	
			Flavonoids:	
			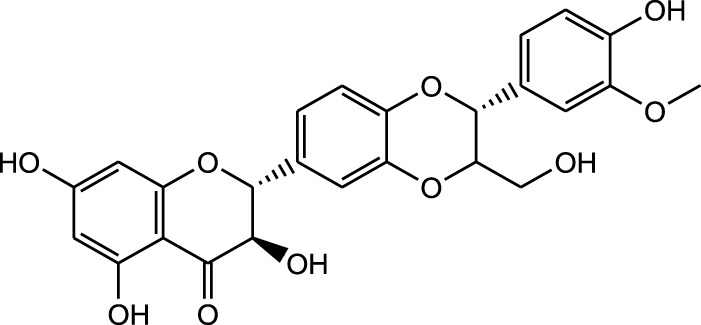 Silybin	
			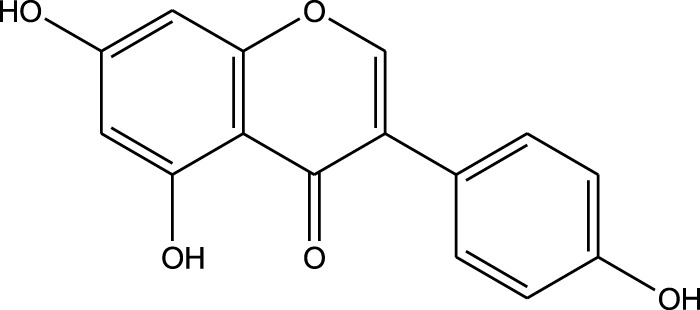	
			Genistein	
Roots				
Methanolic extract	^1^H NMR, ^13^C NMR, and MS	Isolation of 6 pyrrolizidine alkaloids, a carboxylic acid, and phenolic glycosides	Alkaloids:	Braca et al. (2003)
			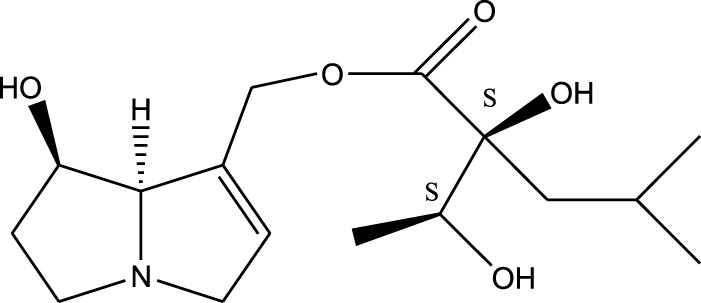 Retronecine 2*S*-hydroxy-2*S*-(1*S*-hydroxyethyl)-4-methyl-pentanoyl ester (PA1)	
			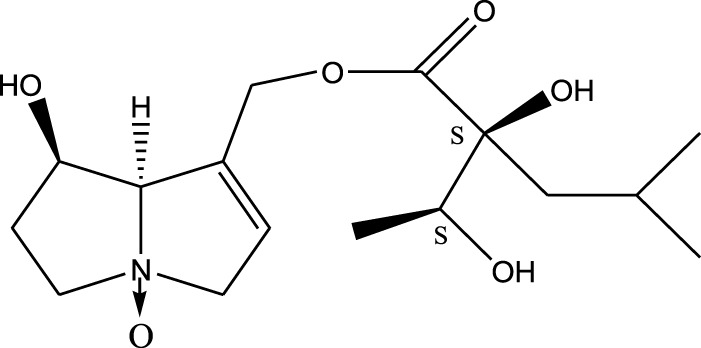	
			Retronecine N-oxide 2*S*-hydroxy-2*S*-(1*S*-hydroxyethyl)-4-methyl-pentanoyl ester (PA2)	
			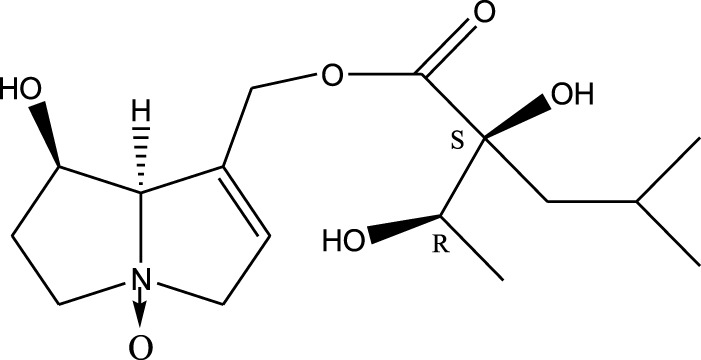	
			Retronecine N-oxide 2*S*-hydroxy-2*S*-(1*R*-hydroxyethyl)-4-methyl-pentanoyl ester (PA3)	
			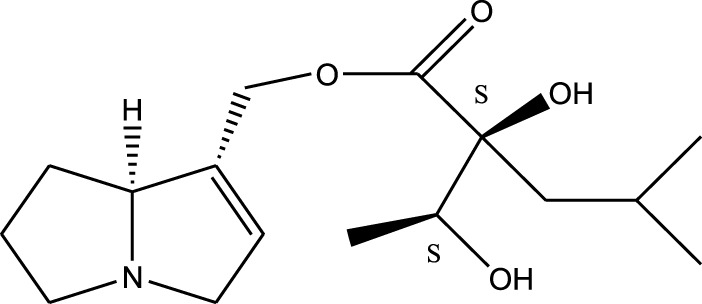	
			Trachelanthamidine 2*S*-hydroxy-2*S*-(1*S*-hydroxyethyl)-4-methyl-pentanoyl ester (PA4)	
			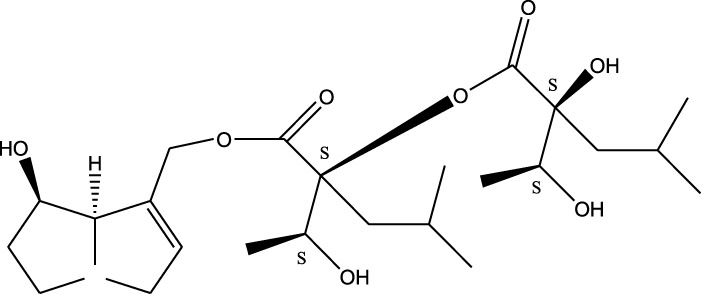	
			Retronecine 2*S*-hydroxy-2*S*-(1*S*-hydroxyethyl)-2*S*-[(1'*S*-hydroxy- ethyl)-4-methylpentanoyl]-4-methylpentanoyl ester (PA5)	
			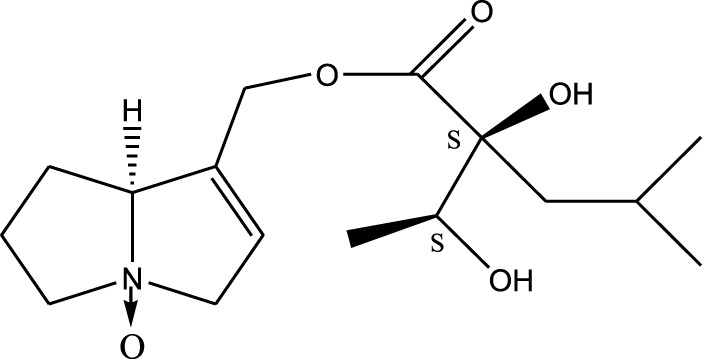	
			Supinidine N-oxide 2*S*-hydroxy-2*S*-(1*S*-hydroxyethyl)-4-methyl-pentanoyl ester (PA6)	
			Phenols:	
			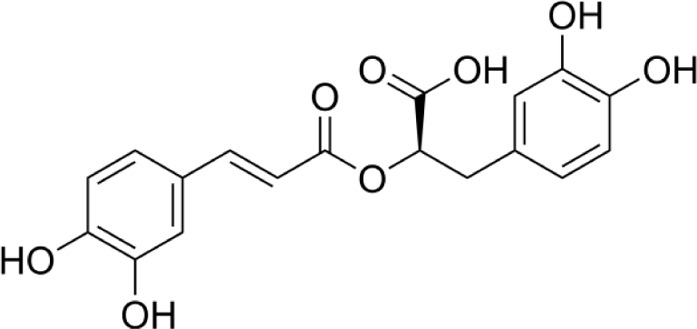 Rosmarinic acid	
			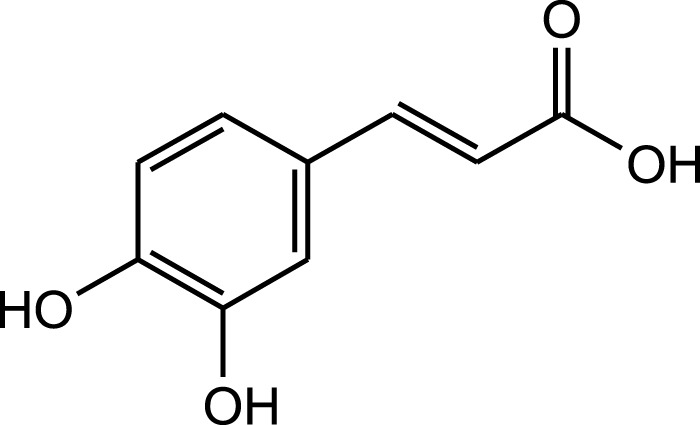	
			Caffeic acid	
			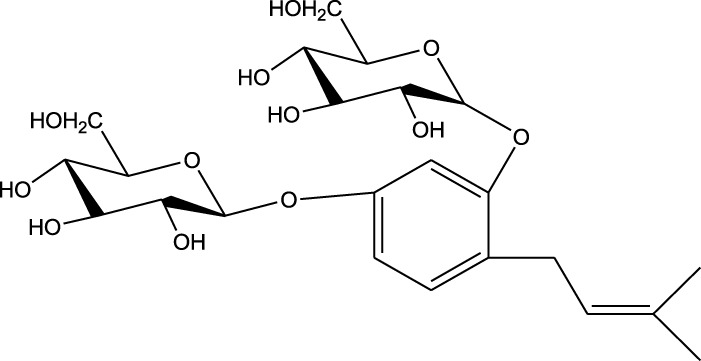	
			1,5-Bis(β-D-glucopyranosyloxy-2-(3',3'-dimethylallyl) benzene	
			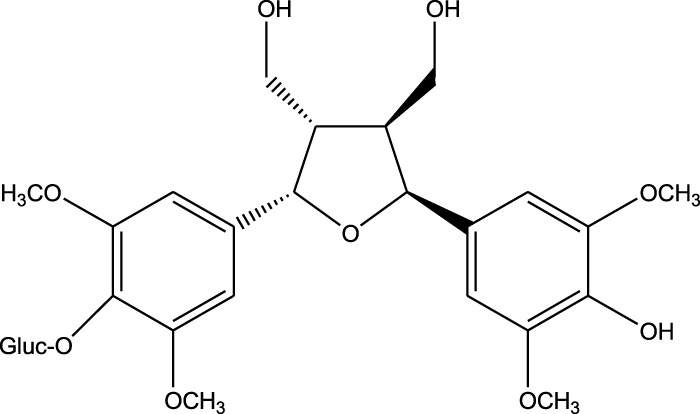	
			7,7′-bis-(4-hydroxy-3,5-dimethoxyphenyl)-8,8′-dihydroxymethyltetrahydrofuran 4′-O-β-D-glucopyranoside	
			Triterpenoids:	
			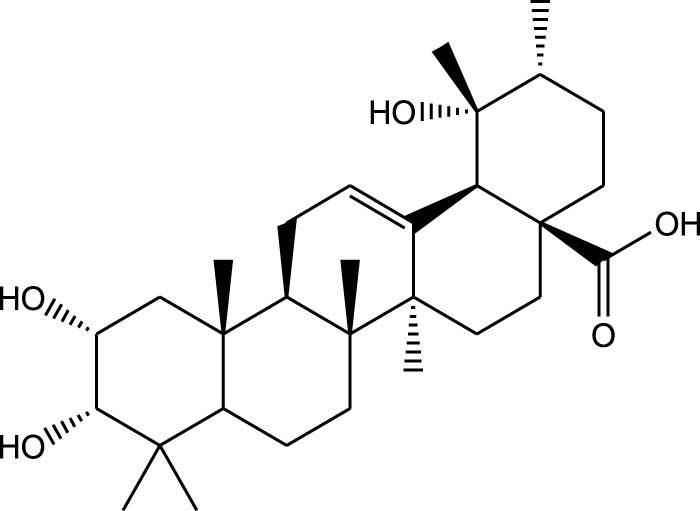 Euscaphic acid	
			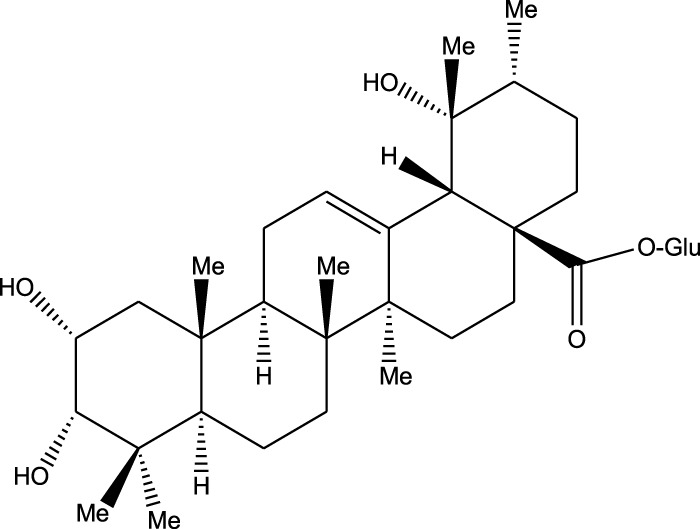	
			Euscaphic acid 28-O-β-D-glucopyranoside	
			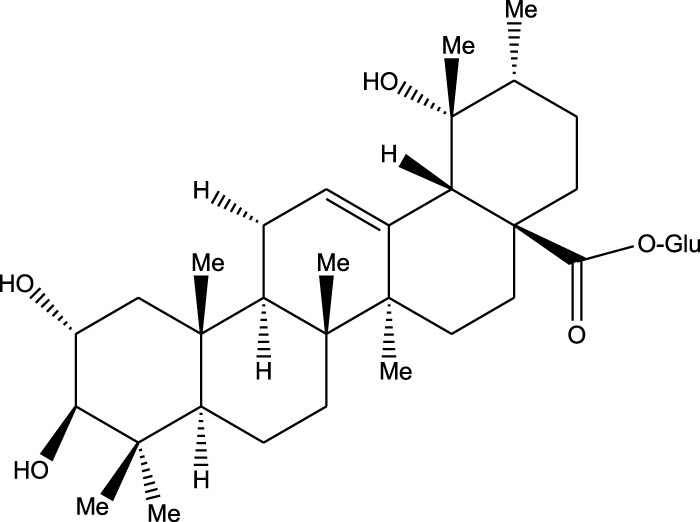	
			Tormentic acid 28-O-β-D-glucopyranoside	
			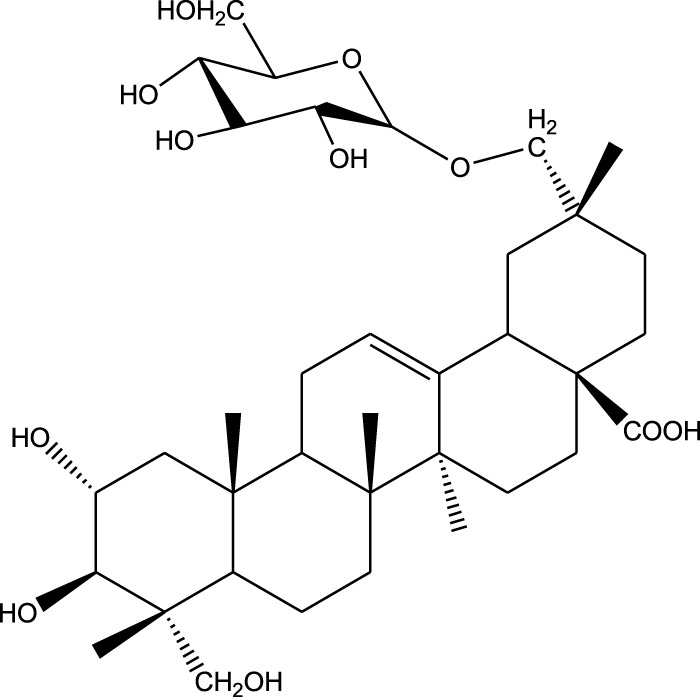	
			2α,3β,23,29-Tetrahydroxyolean-12-en-28-oic acid 29-O-β-D-glucopyranoside	
			Carboxylic acids:	
			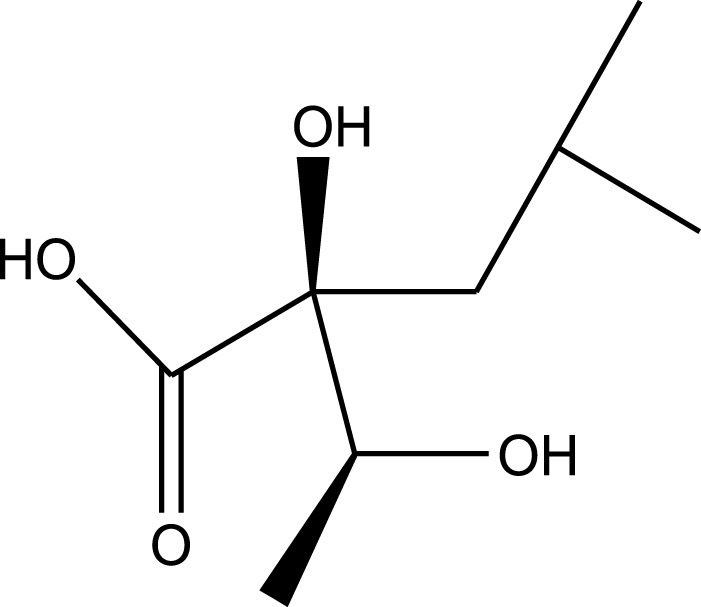 Erythro-2-Hydroxy-2-(1-hydroxyethyl)-4-methyl-pentanoic acid	
			Imidazoles:	
			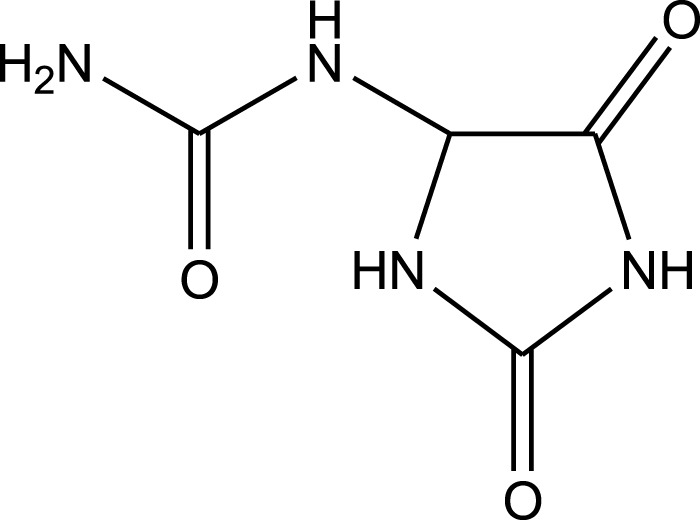	
			Allantoin	
Ethanolic extract	U.V, I.R, ^13^C NMR, and MS	Isolation of 4 triterpenoids with anti-ulcer activity	Triterpenoids:	Abbas et al. (2009)
			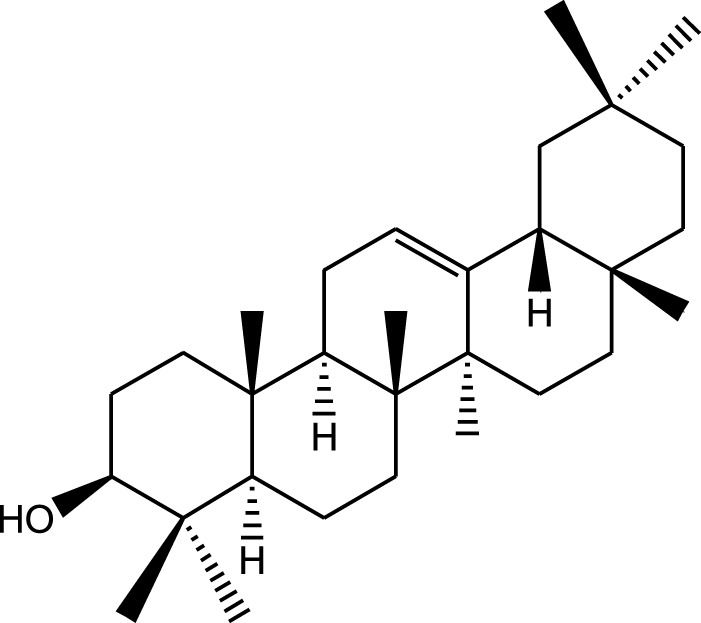	
			β-amyrin	
			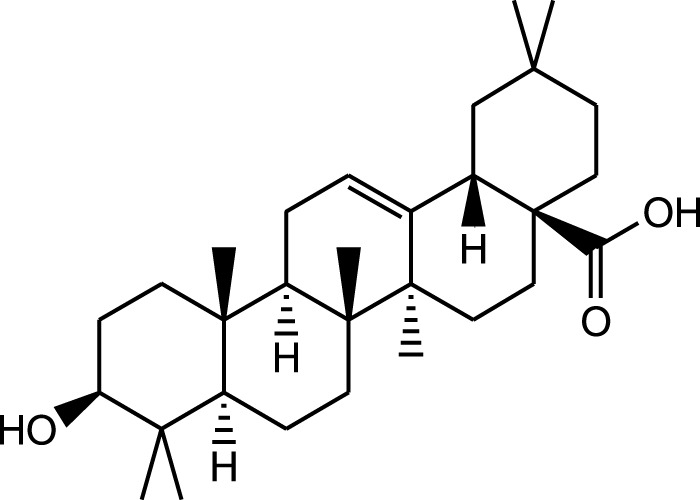	
			Oleanolic acid	
			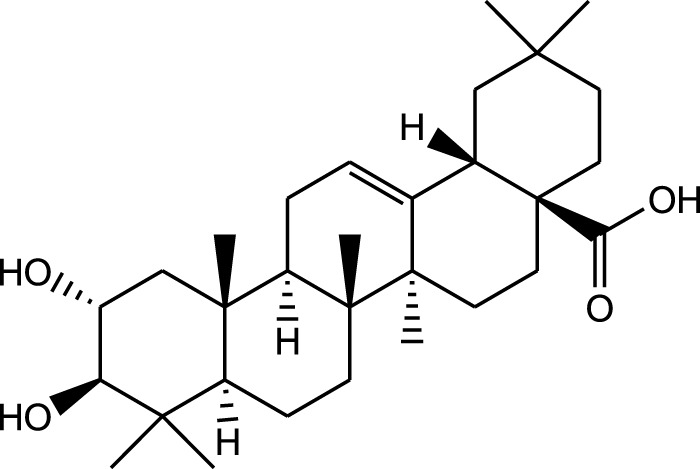	
			Crataegolic acid	
			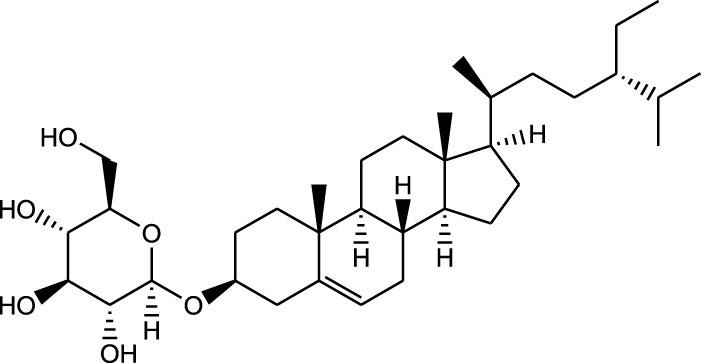	
			β-sitosteryl glucoside	
Methanolic extract	ESI– LC–MS, ^1^H NMR, ^13^C NMR	Isolation of 5 pyrrolizidine alkaloids with antifeeding activity	Alkaloids:	Siciliano et al. (2005)
			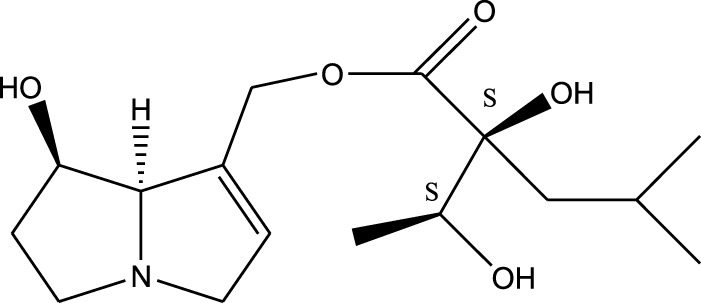 Retronecine 2*S*-hydroxy-2*S*-(1*S*-hydroxyethyl)-4-methyl-pentanoyl ester (PA1)	
			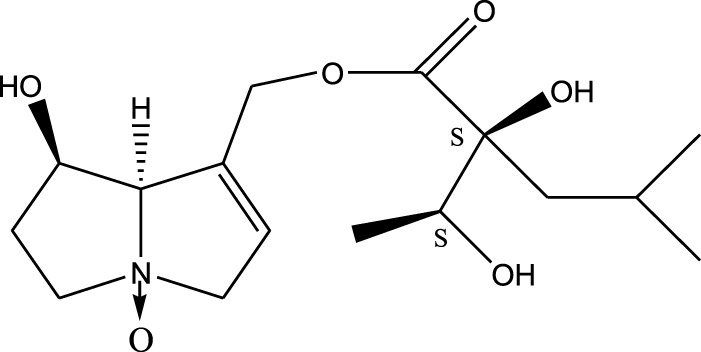	
			Retronecine N-oxide 2*S*-hydroxy-2*S*-(1*S*-hydroxyethyl)-4-methyl-pentanoyl ester (PA2)	
			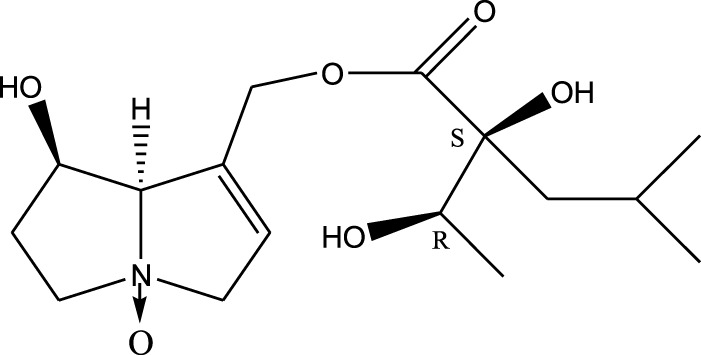	
			Retronecine N-oxide 2*S*-hydroxy-2*S*-(1*R*-hydroxyethyl)-4-methyl-pentanoyl ester (PA3)	
			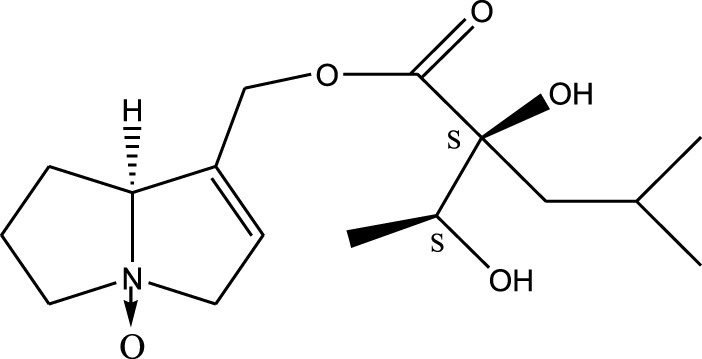	
			Supinidine N-oxide 2*S*-hydroxy-2*S*-(1*S*-hydroxyethyl)-4-methyl-pentanoyl ester (PA6)	
			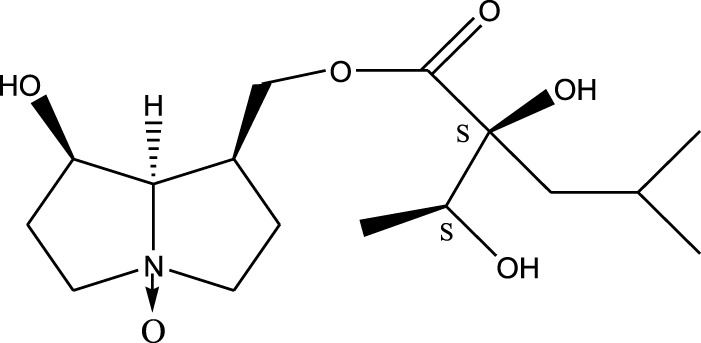	
			Platynecine N-oxide 2*S*-hydroxy-2*S*-(1*S*-hydroxyethyl)-4-methyl-pentanoyl ester (PA8)	
Leaves				
Methanolic extract	ESI– LC–MS, ^1^H NMR, ^13^C NMR	Isolation of 6 pyrrolizidine alkaloids with antifeeding activity	Alkaloids:	Siciliano et al. (2005)
			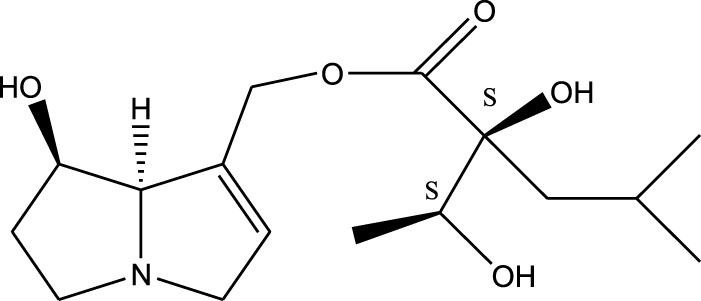 Retronecine 2*S*-hydroxy-2*S*-(1*S*-hydroxyethyl)-4-methyl-pentanoyl ester (PA1)	
			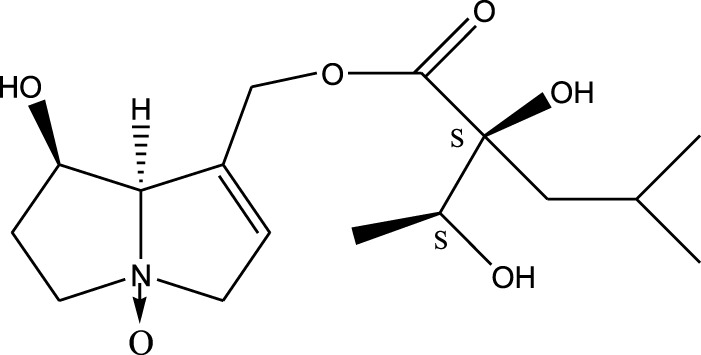	
			Retronecine N-oxide 2*S*-hydroxy-2*S*-(1*S*-hydroxyethyl)-4-methyl-pentanoyl ester (PA2)	
			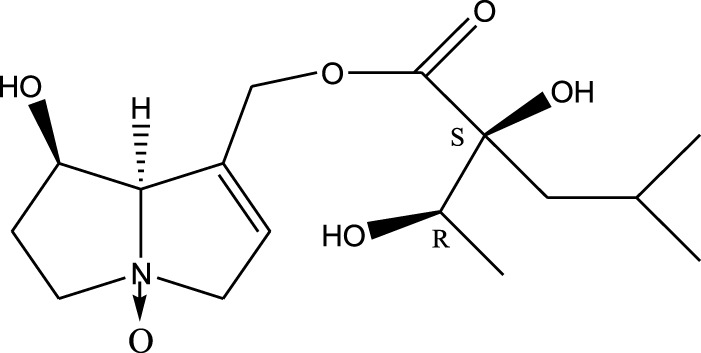	
			Retronecine N-oxide 2*S*-hydroxy-2*S*-(1*R* -hydroxyethyl)-4-methyl-pentanoyl ester (PA3)	
			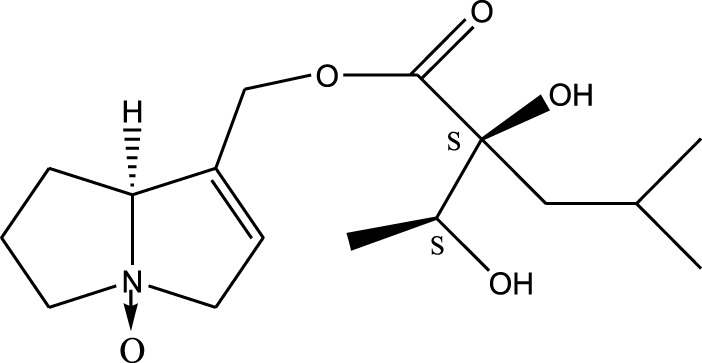	
			Supinidine N-oxide 2*S*-hydroxy-2*S*-(1*S*-hydroxyethyl)-4-methyl-pentanoyl ester) (PA6)	
			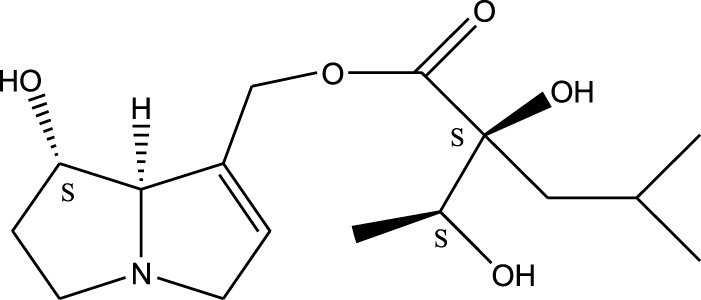	
			Heliotridine 2*S*-hydroxy-2*S*-(1Shydroxyethyl)-4-methyl-pentanoyl ester (PA7)	
			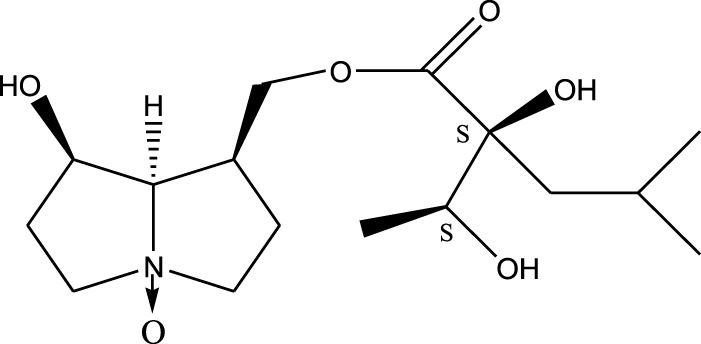	
			Platynecine N-oxide 2*S*-hydroxy-2*S*-(1*S*-hydroxyethyl)-4-methyl-pentanoyl ester (PA8)	
Aqueous extract	HPLC-PDA-MS/MS	Identification of 39 metabolites, rosmarinic acid was the major one, with potential anticancer activity	Phenols:	Chebaro et al. (2023)
			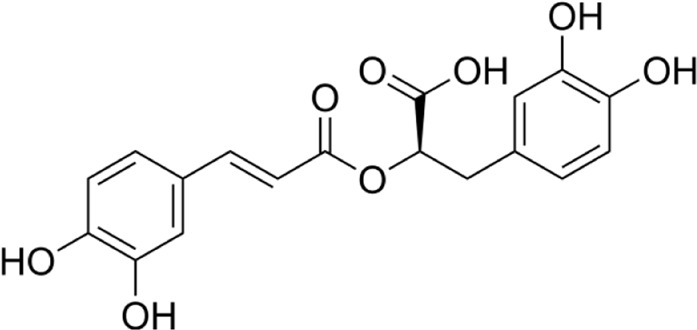 Rosmarinic acid	
			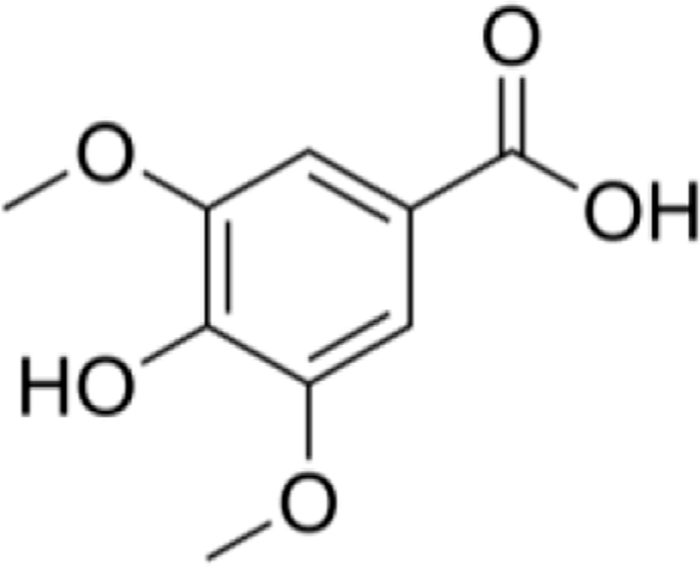	
			Syringic acid	
			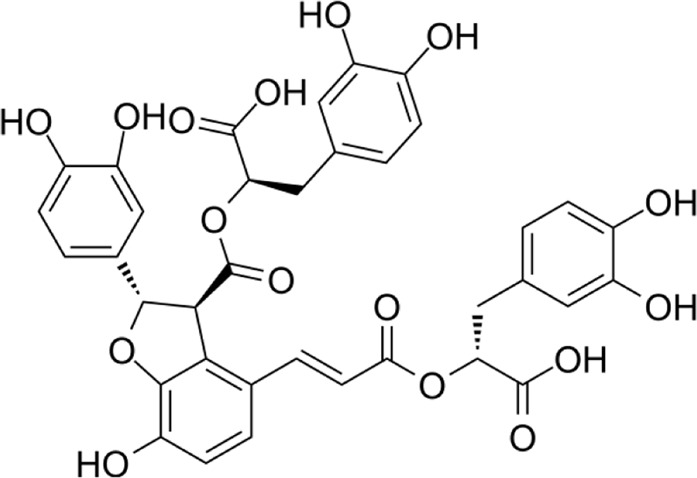	
			Salvianolic acid	
			Flavonoids:	
			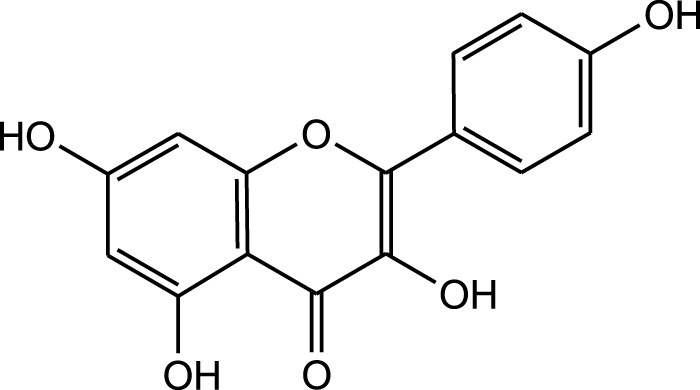 Kampferol	
Flowers				
Methanolic extract	ESI– LC–MS, ^1^H NMR, ^13^C NMR	Isolation of 5 pyrrolizidine alkaloids with antifeeding activity	Alkaloids:	Siciliano et al. (2005)
			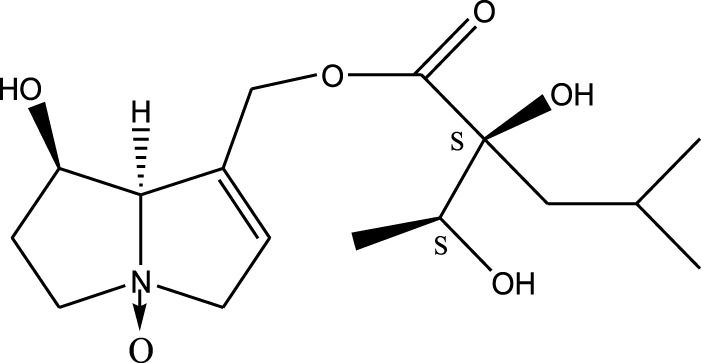	
			Retronecine N-oxide 2*S*-hydroxy-2*S*-(1*S*-hydroxyethyl)-4-methyl-pentanoyl ester (PA2)	
			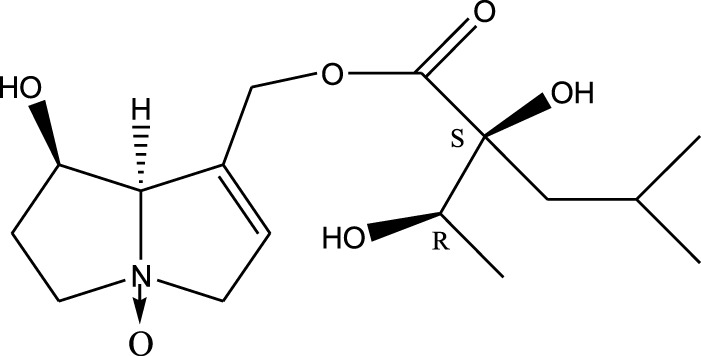	
			Retronecine N-oxide 2*S*-hydroxy-2*S*-(1*R*-hydroxyethyl)-4-methyl-pentanoyl ester (PA3)	
			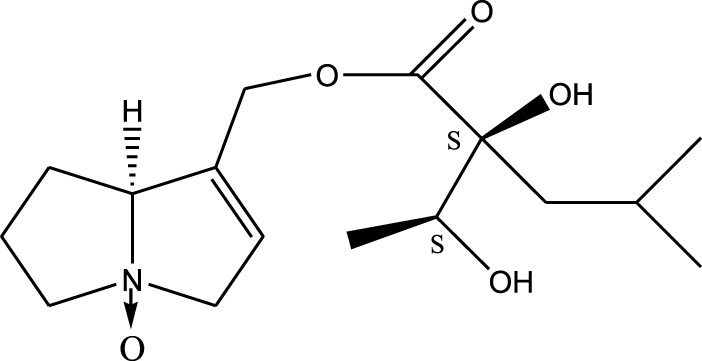	
			Supinidine N-oxide 2*S*-hydroxy-2*S*-(1*S*-hydroxyethyl)-4-methyl-pentanoyl ester) (PA6)	
			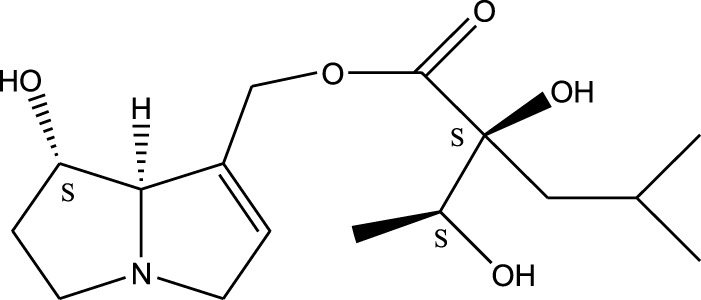	
			Heliotridine 2*S*-hydroxy-2*S*-(1*S*-hydroxyethyl)-4-methyl-pentanoyl ester (PA7)	
			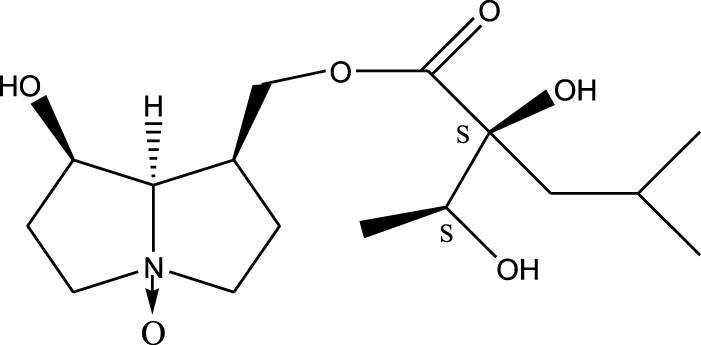	
			Platynecine N-oxide 2*S*-hydroxy-2*S*-(1*S*-hydroxyethyl)-4-methyl-pentanoyl ester (PA8)	
n-hexane extract	2D TLC, GC	Isolation of lipids with antimicrobial activity	Phospholipids:	Al-Salihi F. et al. (2007)
			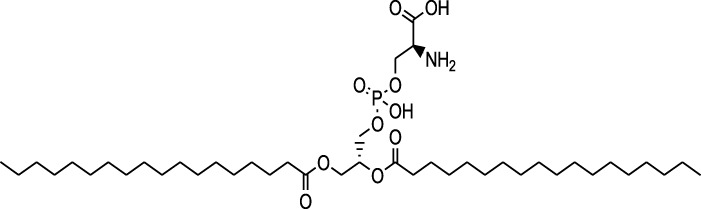	
			Phosphatidylserine	
			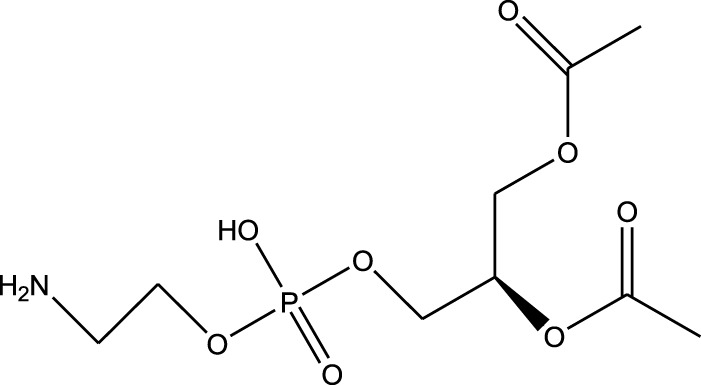	
			Phosphatidylethanolamine	
			Triglycerides:	
			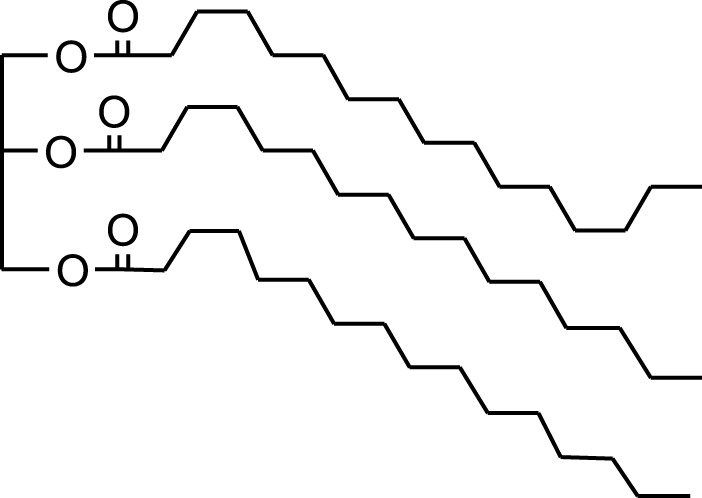 Tripalmitin	
			Fatty acids:	
			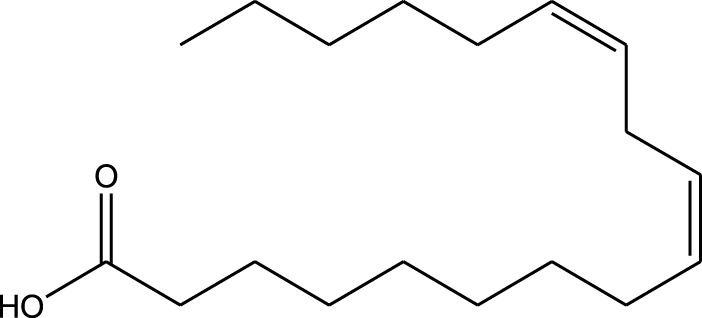	
			Linoleic acid	
			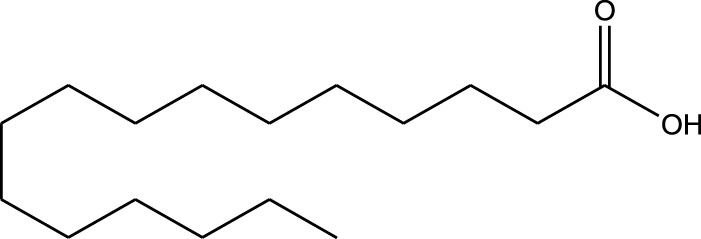	
			Palmitic acid	
Leaves and flowers				
Methanolic extract	HPLC, LC-ESI-MS, MALDI-TOF-MS, GC/MS	Identification of 12 metabolites with pro-wound healing and antimicrobial properties	Flavonoids:	Yarmolinsky et al. (2022)
			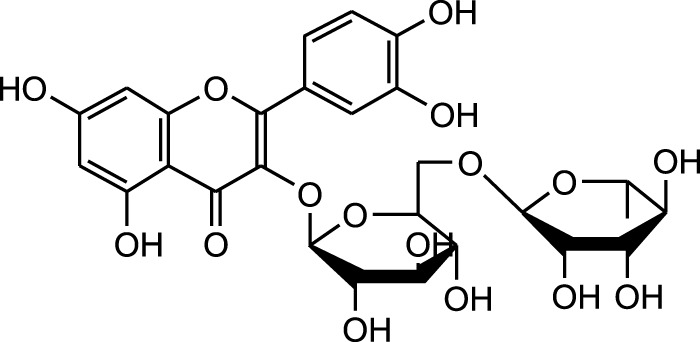	
			Quercetin 3-O-rutinoside	
			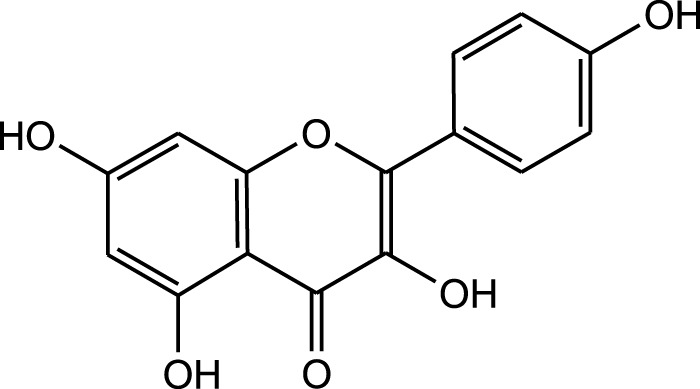	
			Kampferol	
			Aldehydes:	
			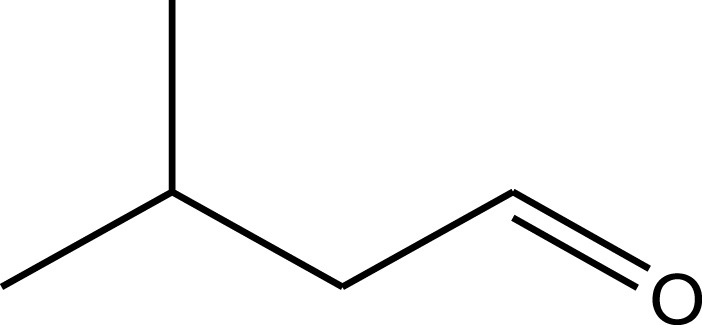	
			3-methylbutanal	
			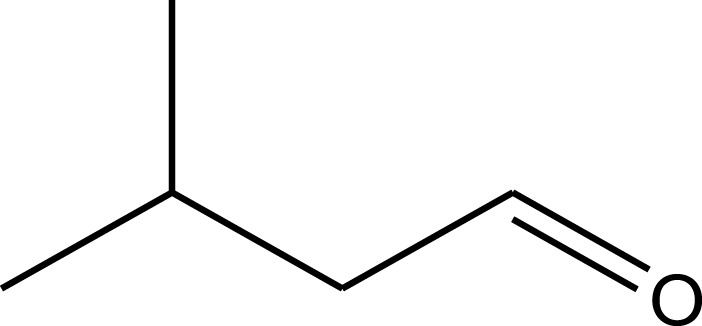	
			Isovaleraldehyde	

A comparison between the phytochemical composition of the different parts of the *A. strigosa* plant shows that the leaves are particularly rich in phenolic acids, flavonoids, and PAs, while the roots were particularly rich in phenolic glycosides, PAs, and triterpenes (Table 3). Moreover, the flowers are rich in fixed and volatile oils, constituting 4.4% of the dry weight, with fixed oils and volatile oils comprising 52.8% and 26.1% of the total lipid content, respectively (Al-Salihi F. et al., 2009). Moreover, the phytochemical composition of plant materials varies with the extraction process and the type of the solvent used, often leading to the identification of different bioactive metabolites. For instance, in a study investigating the composition of *A. strigosa* leaves, the authors showed that 38 metabolites were identified in the aqueous extract obtained by maceration, whereas 39 metabolites were detected from the ultrasound-assisted technique (Chebaro et al., 2023). More importantly, the amount of the major phytochemical metabolites with potent well-documented anticancer activities such as rosmarinic acid, syringic acid, and kaempferol, were present in higher amounts in the maceration extract, and consequently conferred more potent biological activities (Chebaro et al., 2023). Some of the major phytochemicals found in *A. strigosa* are listed in Table 3.

#### 4.3.2 Antioxidant capacity

Oxidative stress is identified as the major cause for the development and progression of aging and several pathophysiological conditions such as cancer*,* inflammation, and neurodegenerative disorders. It is caused by an imbalance between the production and elimination of reactive oxygen species (ROS) and free radicals. The accumulation of these very reactive molecules causes cellular damage because of their unpaired electrons. The levels of ROS and free radicals are normally regulated by endogenous antioxidants or boosted by exogenous sources such as natural plant products. In fact, plants have the ability to synthesize a wide range of phytochemicals that are known to possess potent antioxidant effects that counteract the toxic effects of free radicals. Moreover, natural antioxidant metabolites are regarded as safer alternatives than synthetic ones such as butyl hydroxy anisole (BHA) and butylated hydroxytoluene (BHT), which are widely used as preservatives in food, cosmetic formulations, and other consumer products, and unfortunately associated with potentially toxic and carcinogenic effects (Malkinson, 1983; Lourenço et al., 2019; Felter et al., 2021).

The antioxidant potential of *A. strigosa* has been investigated by using different solvents for the extraction of plant material and assessed through various assays, as tabulated in Table 4. Both aqueous and methanolic extracts showed significant scavenging activities of the 2,2'-azinobis-(3-ethylbenzothiazoline-6-sulfonic acid) (ABTS) radical, which was attributed to the total phenolic content of the extracts (Alali et al., 2007). Similar results were obtained with a methanolic extract from *A. strigosa* flowers using the DPPH radical scavenging and β-carotene bleaching (BCB) assays (Al-Khateeb et al., 2019), as well as the aqueous and hydro-ethanolic extracts of *A. strigosa* aerial parts using the DPPH and ferric reducing power (FRAP) assays (Dibas et al., 2017). More specifically, the hydro-ethanolic extract exhibited stronger antioxidant activity that was correlated with higher phenolic and flavonoid contents. The antioxidant capacity of the leaves was also assessed (Chebaro et al., 2023). Results showed that the aqueous extracts exhibited strong free-radical scavenging activity, which was associated with the presence of secondary metabolites with antioxidant properties. Indeed, the extract showed high concentrations of phenolics, flavonoids, and other metabolites that may be responsible for the observed effect. It is important to mention that the antioxidant capacity of *A. strigosa* has been assessed through *in vitro* assays, which are often prone to errors due to the chemical diversity of phytochemicals. Result should be cautiously interpreted and supported by additional evidence of their beneficial antioxidant use *in vivo*.

**TABLE 4:** The antioxidant capacity of *Anchusa strigosa*.

Extract	Dose	Methods	Observations	References
Aqueous and methanolic extracts of whole plant	Not specified	ABTS Radical Scavenging assay	- Both extracts showed strong activity, with the aqueous extract exhibiting a stronger effect associated with a higher phenolic content	Alali et al. (2007)
Methanolic extract of flowers	Not specified	DPPH radical scavenging assay	- Showed moderate activity	Al-Khateeb et al. (2019)
Methanolic extract of flowers	Not specified	β-carotene bleaching (BCB) assay	- Showed strong activity	Al-Khateeb et al. (2019)
Aqueous and hydro-ethanolic extracts of aerial parts	5–2,500 μg/ml	DPPH radical scavenging assay	- Hydro-ethanolic extract showed strong activity	Dibas et al. (2017)
Aqueous and hydro-ethanolic extracts of aerial parts	0.25–50 mg/mL	Ferric reducing power (FRAP) assay	- Both extracts showed strong activity	Dibas et al. (2017)
Aqueous extract of leaves	100–1,000 μg/mL	DPPH radical scavenging assay	- Showed strong activity	Chebaro et al. (2023)

## 5 Biological activities of *Anchusa strigosa*

**FIGURE 2:**
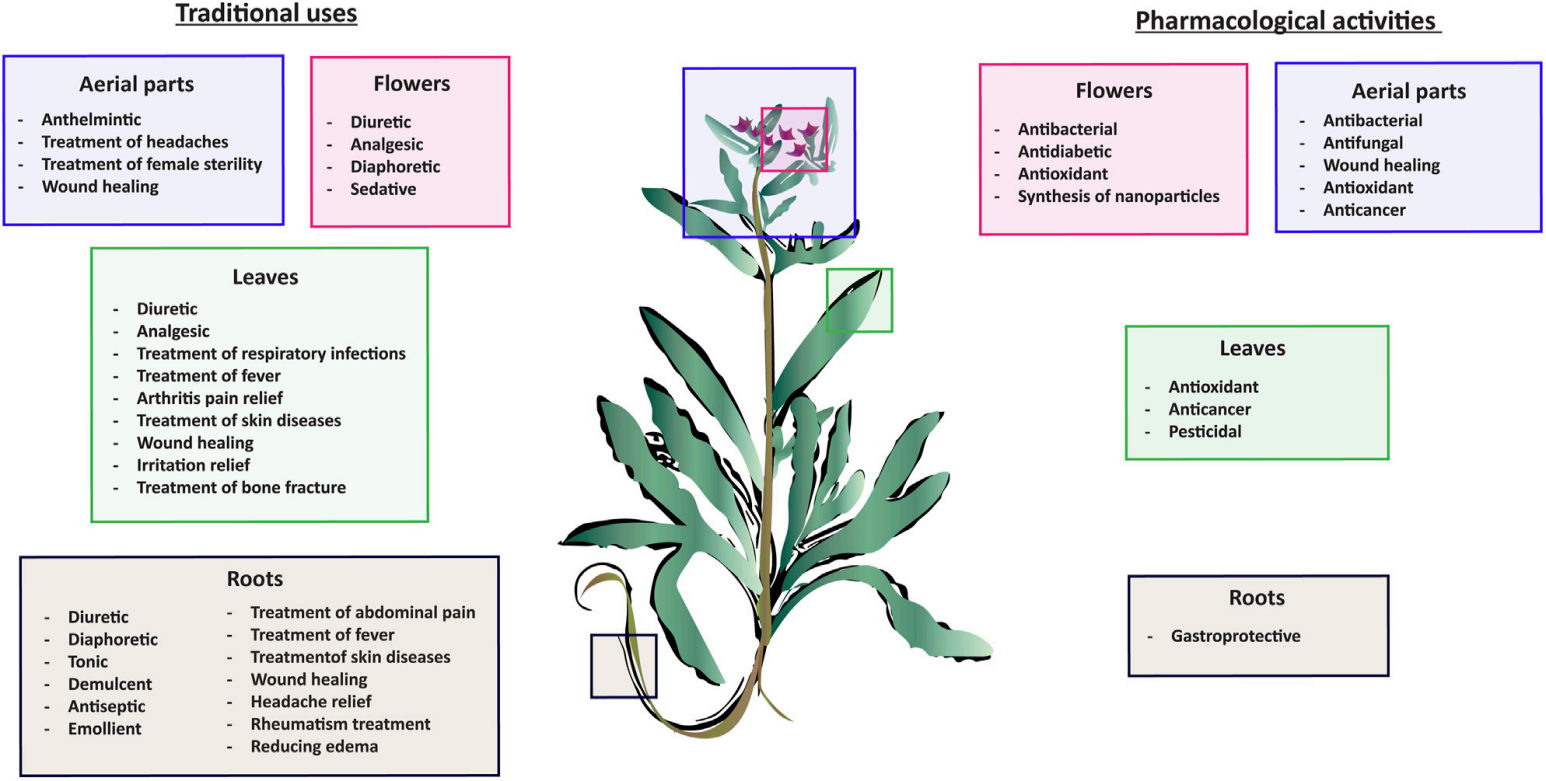
Summary of the main traditional uses of *Anchusa strigosa* and tested pharmacological activities.

In recent years, several studies demonstrated the vast range of biological and pharmacological properties of *A. strigosa* including antimicrobial, pro-wound healing, antioxidant, anti-inflammatory, anticancer, antiarthritic, gastric protective, and antidiabetic effects. These are summarized in Figure 2.

### 5.1 Antimicrobial activities

Antimicrobial resistance is a major global challenge and a serious threat to humans, which is caused by the extensive and inappropriate use of antibiotics. As traditional drug therapies have been losing their effectiveness, there has been an urgent need to develop new antimicrobial agents using novel therapies based on natural metabolites due to the chemical diversity and efficacy. In fact, extracts from medicinal plants have been widely reported to exhibit antimicrobial activities. They act by inhibiting the growth of a variety of pathogens, including bacteria, fungi, and viruses, in addition to enhancing the activity of antibiotics, and may help overcome antimicrobial resistance (Vaou et al., 2021). The effectiveness of *A. strigosa* extracts against a broad range of pathogenic microorganisms, including gram-positive and gram-negative bacteria, fungi, yeast, and pests has been extensively analyzed and listed in Table 5.

**TABLE 5:** The antimicrobial effects of *Anchusa strigosa*.

Extract	Dose	Experimental model	Organisms	Main results	References
Antibacterial					
Essential oil and fixed oil of the flowers	Dose range: 10–500 μg/mL Minimal active dose: - Essential oil:200 μg/mL - fixed oil: 500 μg/mL	Method: Agar disk diffusion	*Bacillus subtilis*	- Essential oil was more active than fixed oil - Essential oil showed potent activity specially against *Pseudomouos aeruginsa*, *Proteus sp*. and *Streptococcus faecalis* - Fixed oil showed strong activity against *P. aeruginosa*, *Klebsiella* sp. *and, Proteus* sp.	Al-Salihi F. et al. (2009)
		Positive control:	*Enterobacter* sp.		
		- Pencillin (10 units)	*Escherichia coli*		
		- Ampicillin (10 mg)	*Klebsiella* sp.		
		- Carbenicillin (100 mg)	*Proteus* sp.		
		- Chloramphenicol (30 mg)	*Pseudomouos aeruginsa*		
		- Nitrofurantion (300 mg)	*Staphylococcus aureus Staphyloccus epidermidis*		
		- Nalidixic acid (30 mg)	*Streptococcus faecalis*		
		- Cephalexin (30 mg)	*Streptococcus viridance*		
		- Tetracycline (30 mg)			
		- Kanamycin (30 mg)			
		- Erythromycin (15 mg)			
Total lipids of flowers using n-hexane	Dose range: 0.01–10 mg/mL Minimal active dose: 0.5 mg/mL	Method: Agar disk diffusion	*Bacillus subtilis*	- Strong antibacterial activity. - More effective against gram-positive bacteria. - Most susceptible Gram-positive bacteria was *Streptococcus faecalis*.- Most susceptible gram-negative bacteria was *Pseudomonas aeruoginosa.*	Al-Salihi F. et al. (2007)
		Positive control:	*Enterobacter* sp.		
		- Pencillin (10 units)	*Escherichia coli*		
		- Ampicillin (10 mg)	*Klebsiella sp*.		
		- Carbenicillin (100 mg)	*Proteus sp*.		
		- Chloramphenicol (30 mg)	*Pseudomonas aeruoginosa*		
		- Nitrofurantion (300 mg)	*Staphylococcus aureus*		
		- Nalidixic acid (30 mg)	*Staphylococcus epidermidis*		
		- Cephalexin (30 mg)	*Streptococcus faecalis*		
		- Tetracycline (30 mg)	*Streptococcus viridians*		
		- Kanamycin (30 mg)			
		- Erythromycin (15 mg)			
Ethanolic extract of aerial parts	10 mg/mL	Method: Agar disk diffusion	*Escherichia coli*	- Strong antibacterial activity against *Staphylococcus aureus*	Ali-Shtayeh et al. (1998)
		Positive control:	*Klebsiella pneumonia*		
		- Ampicillin	*Proteus vulgaris*		
		- Penicillin-G	*Pseudomonas aeruginosa Staphylococcus aureus*		
		- Gentamicin			
Ethanolic extract of leaves and flowers	0.1 mg/mL	Method: WST-1 assay	*Acinetobacter baumannii*	- Strong activity against drug-resistant bacteria	Yarmolinsky et al. (2022)
			*Escherichia coli*		
			*Klebsiella pneumoniae*		
			*Salmonella enteritidis Serratia marcescens*		
Aqueous extract of aerial parts	10 mg/mL	Method: Agar disk diffusion	*Escherichia coli*	- Good antibacterial activity against *Proteus vulgaris*	Ali-Shtayeh et al. (1998)
		Positive control:	*Klebsiella pneumonia*		
		- Ampicillin	*Proteus vulgaris*		
		- Penicillin-G	*Pseudomonas aeruginosa Staphylococcus aureus*		
		- Gentamicin			
Aqueous extract of stems, leaves, and roots	Not specified	Method: Agar disk diffusion	*Aeromonas hydrophila*	- Strong antibacterial effect against *Photobacterium damselae*	Abutbul et al. (2005)
			*Photobacterium damselae*		
			*Streptococcus iniae*		
			*Vibrio alginolyticus*		
Antifungal					
Ethanolic extract of aerial parts	10 mg/mL	Method: Agar disk diffusion	*Candida albicans*	- Good antifungal activity	Ali-Shtayeh et al. (1998)
		Positive control:			
		- Nystatin			
Aqueous extract of aerial parts	15 μg/mL	Method: Agar dilution	*Microsporum canis*	- Good inhibitory effect against the three tested dermatophytes	Ali-Shtayeh and Abu Ghdeib (1999)
		Positive control:	*Trichophyton mentagrophytes*		
		- Griseofulvin	*Trichophyton violaceum*		

Results showed that lipid extracts from *A. strigosa* flowers exhibit potent antimicrobial activity, particularly against gram-positive bacteria such as *Streptococcus faecalis* and *Staphylococcus aureus* and against the gram-negative bacteria *Pseudomouos aeruginsa*, (Al-Salihi F. et al., 2007, 2009). Further analysis of the total lipids extract showed the presence of two phospholipids (phosphatidyl serine and phosphatidylethanolamine) and a triglyceride (tripalmitin) (Al-Salihi F. et al., 2007). Moreover, the authors showed that the essential oil extract from *A. strigosa* flowers showed stronger activity than the fixed oil extract (Al-Salihi F. et al., 2009). Several studies demonstrated the antimicrobial effects of *A. strigosa* ethanolic extracts. For example, Ali-Shtayeh et al. (1998) demonstrated its effect against the gram-positive bacteria *Staphylococcus aureus*, as well the yeast *Candida albicans*. And Yarmolinsky et al. (2022) showed that the crude extract as well as the isolated metabolite kaempferol and its glycoside derivatives significantly inhibited the growth of gram-negative bacteria that show drug-resistance such as *Escherichia coli*, *Klebsiella pneumoniae*, *Acinetobacter baumannii*, *Serratia marcescens*, and *Salmonella enteritidis*. Aqueous extracts also showed potent antimicrobial activity against the gram-negative bacteria *Proteus vulgaris* (Ali-Shtayeh et al., 1998) and bacterial pathogens that infect fish such as *P. damselae* (Abutbul et al., 2005), as well as antifungal activity against *Microsporum canis*, *Trichophyton mentagrophytes*, and *Trichophyton violaceum* (Ali-Shtayeh and Abu Ghdeib, 1999).

### 5.2 Antifeedant effect

Plant extracts have long been shown to exhibit potent antifeeding activities against insects. PAs isolated from the methanolic extract of *A. strigosa* leaves showed potent antifeedant activity against the generalist beet armyworm *Spodoptera exigua* and the *Pieris brassicae* specialist larvae (Siciliano et al., 2005). Interestingly, only 1,2-unsaturated PAs showed antifeedant activity against the tested pests, further supporting their high toxic potential.

### 5.3 Wound healing properties

**FIGURE 3:**
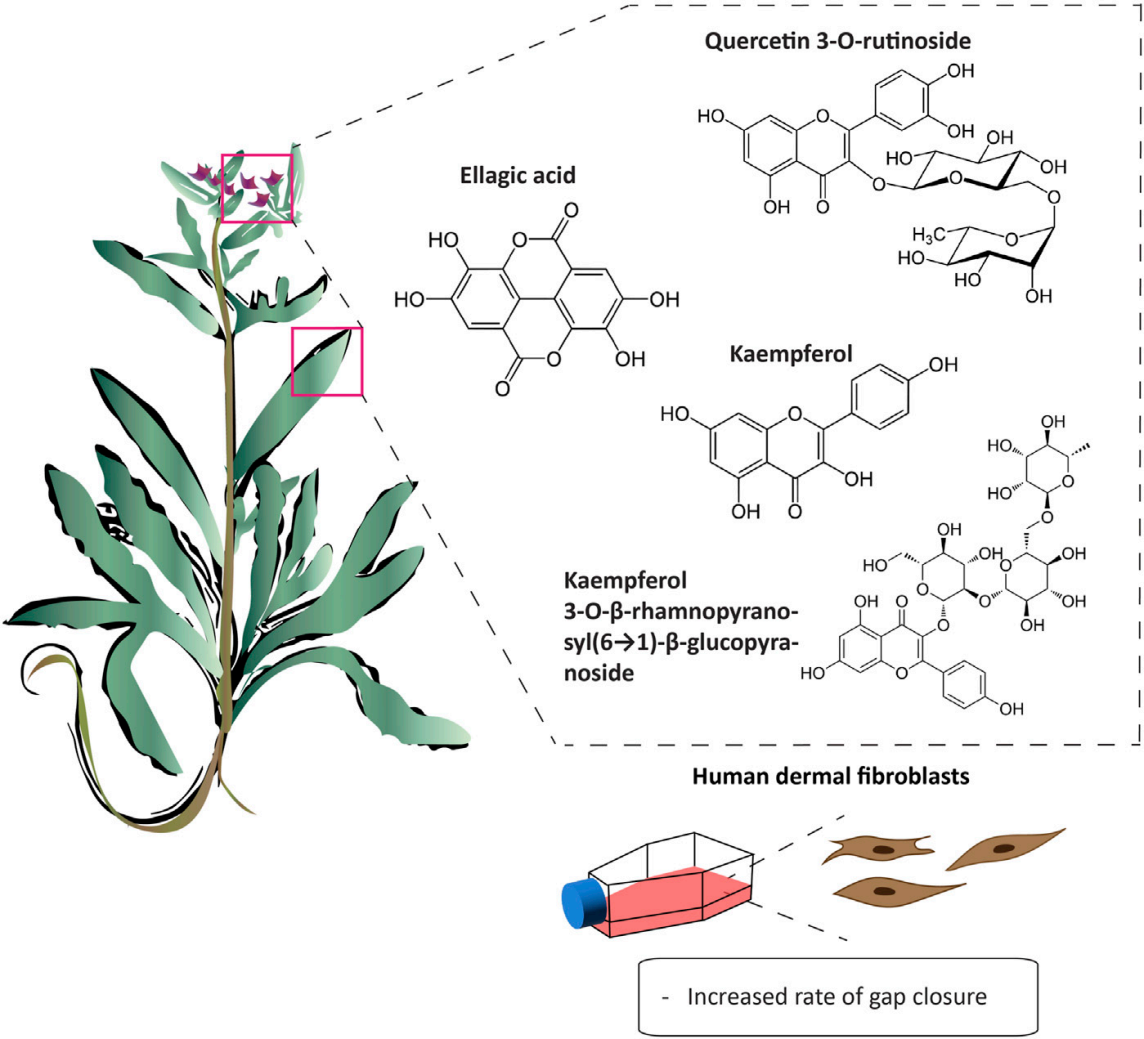
*Anchusa strigosa* exhibits wound healing properties. *A. strigosa* stimulates wound healing by increasing the rate of gap closure in cultured human dermal fibroblast cells. This is due to the synergistic action between metabolites isolated from the extract, with quercetin 3-O-rutinoside, ellagic acid, kaempferol, and kaempferol 3-O-β-rhamnopyranosyl(1→6)-β- as the main wound healing components.

Wound healing is dynamic process that is characterized by four major stages: homeostasis, inflammation, proliferation, and remodeling. Each of these stages is driven by biological and chemical processes that protect the area from infection and lead to the regeneration of damaged tissue. *A. strigosa* has been widely used in traditional medicine for the treatment of topical wounds. As such, the mechanism of action behind the pro-wound healing activity of *A. strigosa* leaves and flowers was investigated in a study using a human dermal fibroblasts cell line (Yarmolinsky et al., 2022). Results showed that the crude methanolic extract significantly stimulated wound healing by increasing the rate of gap closure in the cultured cells. Moreover, it is the synergistic action between the isolated metabolites that confers the plant extract its pro-wound healing property. The authors identified quercetin 3-O-rutinoside, ellagic acid, kaempferol, and kaempferol 3-O-β-rhamnopyranosyl(1→6)-β-glucopyranoside as the pro-wound healing metabolites of the extract, calling for further investigation behind the mechanism of action of the tested metabolites. This is presented in Figure 3 and Table 6. While the *in vitro* human dermal fibroblast model provides a quick and inexpensive method to screen for the pro-wound healing property of an extract, it mainly focuses on the proliferation and migration of fibroblasts and does not assess other factors in the wound healing process. More importantly, the safety profile of the extract should be further investigated using *in vivo* studies by assessing its effect through systemic (oral or parenteral) and topical administrations.

**TABLE 6:** The pharmacological effects of *Anchusa strigosa*.

Extract	Dose	Experimental model	Observations	References
Antifeedant				
Pyrrolizidine alkaloids isolated from *A. strigosa*	Dose range: 0.01–10 μg/cm^2^	Method:Free choice feeding assay	- Concentration-dependant antifeedant activity against *Pieris brassicae and Spodoptera exigua*	Siciliano et al. (2005)
Pro-wound healing				
Methanolic extract of leaves and flowers	50 μg/mL	Method: Scratch assay on human dermal fibroblasts cell line (*in vitro* wound healing model)	- Significant stimulation of wound healing - Crude extract had a stronger pro-wound healing activity than each identified metabolite alone	Yarmolinsky et al. (2022)
Anticancer				
Hydro-ethanolic extract of aerial parts	Dose range: 0–200 μg/mL IC50: - 252 μg/mL (SW480) - 186 μg/mL (HTC116)	Method: *in vitro* MTT cell viability assay on human colorectal cancer cell lines (SW480 and HCT116)	- Showed the strongest anti-proliferative activity among tested plants	Dibas et al. (2017)
Extracts of leaves or roots obtained by different solvents	Dose range: 3.9–250 μg/mL	Method: *in vitro* SRB cell viability assay on colorectal cancer (Caco-2) and breast cancer (T-47D, MDA-MB-231, and MCF-7) cell lines	- Leaves showed stronger anti-proliferative activity than the roots	Al-Khatib et al. (2021)
Ethanolic extract of aerial parts	Dose range: 0.01–100 μg/mL	Method: *in vitro* SRB cell viability assay on colorectal cancer (HT-29) and breast cancer (MCF-7) cell lines	- Showed moderate cytotoxicity against both HT-29 and MCF-7 cell lines with IC_50_ values of 188.84 μg/mL ± 12.91 and 191.48 μg/mL ± 5.67 at 72 h, compared to the other tested plants	Alruwad et al. (2023)
	IC50:			
	−188.84 μg/mL (HT-29)			
	−191.48 μg/mL (MCF-7)			
Aqueous extract of leaves	Dose range: 200–400 μg/mL	Method: - *in vitro* MTT cell viability assay on pancreatic ductal carcinoma cell line (Capan-2) - Western blot - Scratch assay	- Significantly inhibited cell proliferation (with IC_50_ values of 2136.6, 404.98, and 370.6 μg/mL at 24, 48, and 72 h). - induced apoptosis, and inhibited cell migration.	Chebaro et al. (2023)
	IC50:			
	−2136.6 μg/mL (24 h)			
	−404.98 μg/mL (48 h)			
	−370.6 μg/mL (72 h)			
Anti-inflammatory				
Aqueous and methanolic extracts of the whole plant	Dose range: 250 and 500 mg/kg Minimal active dose: 250 mg/kg	Method: Complete Freund’s Adjuvant (CFA)-induced arthritis in rats Positive control: - Betamethasone (3 mg/kg)	- Both extracts attenuated paw edema, arthritis index, and hematological abnormalities, in addition to restauration of body weight	Alallan et al. (2018)
Gastroprotective				
Aqueous extract of roots	Dose range: 0.04 and 0.08 g/animal Minimal active dose: 0.04 g/animal	Method: Ethanol-induced gastric ulcer model in rats	- Pre-treatment decreased the ulcer index by 82.4% and 93.2% according to morphometric and planimetric methods, respectively. - Inhibited stomach ulceration in a concentration-dependent manner	Disi et al. (1998)
Aqueous extract of roots	0.286 g/kg body weight/day	Method: Ethanol-induced gastric ulcer model in guinea pigs	- Treatment with the therapeutic dose healed the gastric lesions, resulting in full recovery	Disi et al. (1998)
Ethanolic extract of roots (petroleum ether fraction)	5 ml/kg	Method: Ethanol-induced gastric ulcer model in rats	- Caused 91% inhibition of gastric lesions	Abbas et al. (2009)
Ethanolic extract of roots (chloroform fraction)	5 ml/kg	Method: Ethanol-induced gastric ulcer model in rats	- Caused 86% inhibition of gastric lesions	Abbas et al. (2009)
Ethanolic extract of roots (butanol fraction)	5 ml/kg	Method: Ethanol-induced gastric ulcer model in rats	- Caused 65% inhibition of gastric lesions	Abbas et al. (2009)
Ethanolic extract of roots (aqueous fraction)	5 ml/kg	Method: Ethanol-induced gastric ulcer model in rats	- No significant inhibition	Abbas et al. (2009)
Aqueous extract of roots	Not specified	Method: Pepsin inhibition assay	- Pepsin was inhibited by the crude extract	Abuereish (1998)
Antidiabetic				
Aqueous extract of flowers	Dose range: 250 and 500 mg/kg	Method: Streptozotocin-induced diabetic rat model	- Decreased blood glucose, cholesterol, and triglyceride levels in a dose-dependent manner	Beyatli and Ari (2012)
	Minimal active dose: 250 mg/kg	Positive control: Glibenclamide (0.6 mg/kg)	- Increased serum insulin levels and hepatic glycogen levels in a dose-dependent manner	

### 5.4 Anticancer activities

**FIGURE 4:**
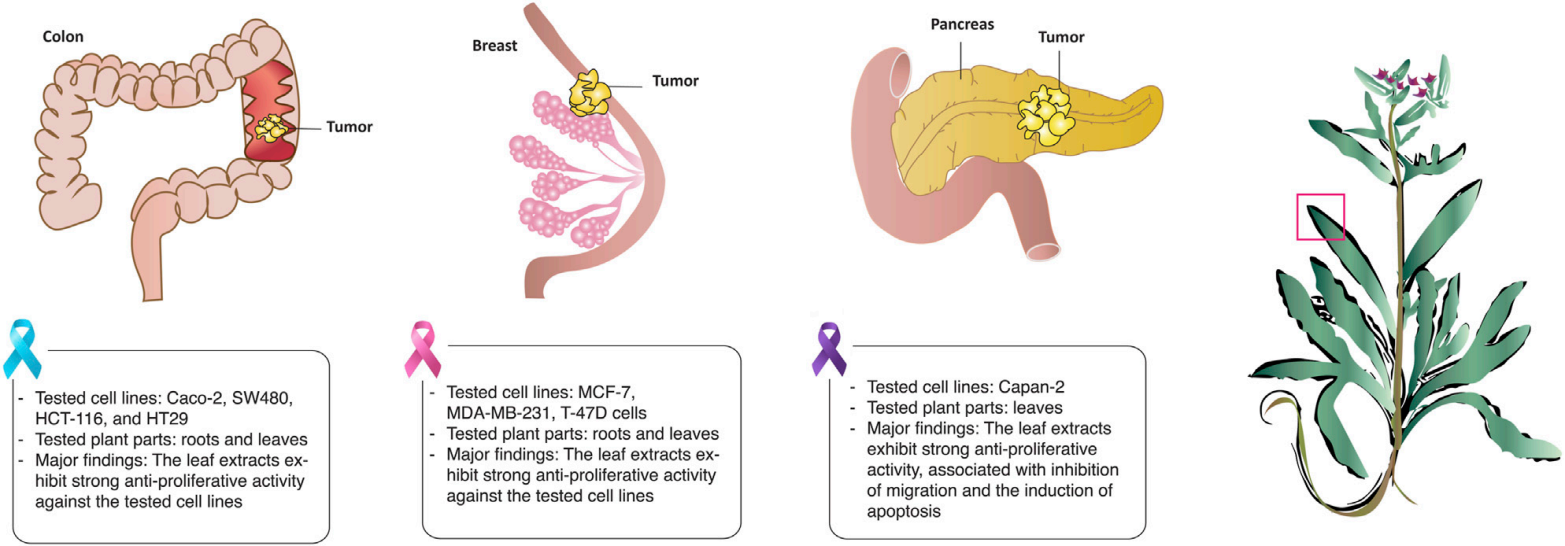
*Anchusa strigosa* shows strong antitumorigenic properties against several cancers. *A. strigosa* leaf extracts reduces the proliferation and growth rate of colorectal, breast, and pancreatic cancer cells *in vitro*.

Cancer is a global health burden and one of the leading causes of mortality worldwide. Despite recent advances in cancer therapy, adverse side effects and multidrug resistance continues to be a major challenge to conventional treatment regimens, which has fueled recent interest in the search for new bioactive metabolites from natural plant sources (Gezici and Şekeroğlu, 2019; Khan et al., 2020). In a study screening different plants in Jordan for their cytotoxicity against human colorectal cancer, the authors showed that the hydro-ethanolic extract of aerial parts of *A. strigosa* showed the strongest anti-proliferative activity among the tested plants with IC_50_ values of 186 and 252 μg/mL against the SW480 and HCT116 cell lines, respectively (Dibas et al., 2017). The anticancer properties of *A. strigosa* extracts from roots and leaves was also tested against several cancer cell lines including the colorectal carcinoma Caco-2, the human breast ductal carcinoma T-47D, the human breast carcinoma MDA-MB-231, and the breast adenocarcinoma MCF-7. Results showed that the leaves exhibited stronger anti-proliferative activity than the roots, probably due to the higher concentration of tannins and PAs (Al-Khatib et al., 2021). The cytotoxicity of the areal parts of *A. strigosa* was further confirmed in a study using an ethanolic extract against the HT-29 colorectal and MCF-7 breast cancer cell lines (Alruwad et al., 2023). Moreover, the aqueous extract of *A. strigosa* leaves showed strong anticancer activity against the aggressive pancreatic ductal carcinoma capan-2 cells (Chebaro et al., 2023). Notably, the extract exhibited potent inhibitory effects on capan-2 cells’ proliferation and migration, along with an induction of cell-cell aggregation and apoptosis. The anticancer properties of *A. strigosa* are shown in Figure 4 and summarized in Table 6. Overall, *A. strigosa* leaves prove to be an effective source of bioactive metabolites with anticancer potential that still warrants further investigation.

### 5.5 Anti-inflammatory and anti-arthritic activities

Inflammation is a natural response by the body to fight an infection or repair an injury (National Institute of Environmental Health Sciences, Inflammation, 2023). However, when inflammation becomes chronic, it may lead to several diseases, including cardiovascular diseases, cancer, diabetes, neurodegenerative diseases, and arthritis. The anti-inflammatory property of *A. strigosa* was investigated first using Complete Freund’s Adjuvant (CFA)-induced paw edema in rats (Alallan et al., 2018). Treatment with the aqueous and methanolic extracts of the whole plant both showed significant reduction in swelling, similar to the betamethasone control, suggesting that *A. strigosa* could be a source of bioactive metabolites involved in the acute inflammatory response. The extracts were further investigated for the anti-arthritic effect, using CFA-induced arthritis in rats, as model for chronic immune-mediated joint inflammation (Alallan et al., 2018). Results showed that the arthritis index was significantly lowered with both extracts. This was accompanied by a restauration of body weight, which was significantly lowered due to CFA treatment, in addition to an attenuation of hematological abnormalities, showing increased levels of hemoglobin and a reduction of elevated white blood cell levels following treatment. These findings are summarized in Table 6. These indicate that *A. strigosa* have potential therapeutic activities in the treatment of rheumatoid arthritis and inflammatory diseases.

### 5.6 Gastroprotective properties

**FIGURE 5:**
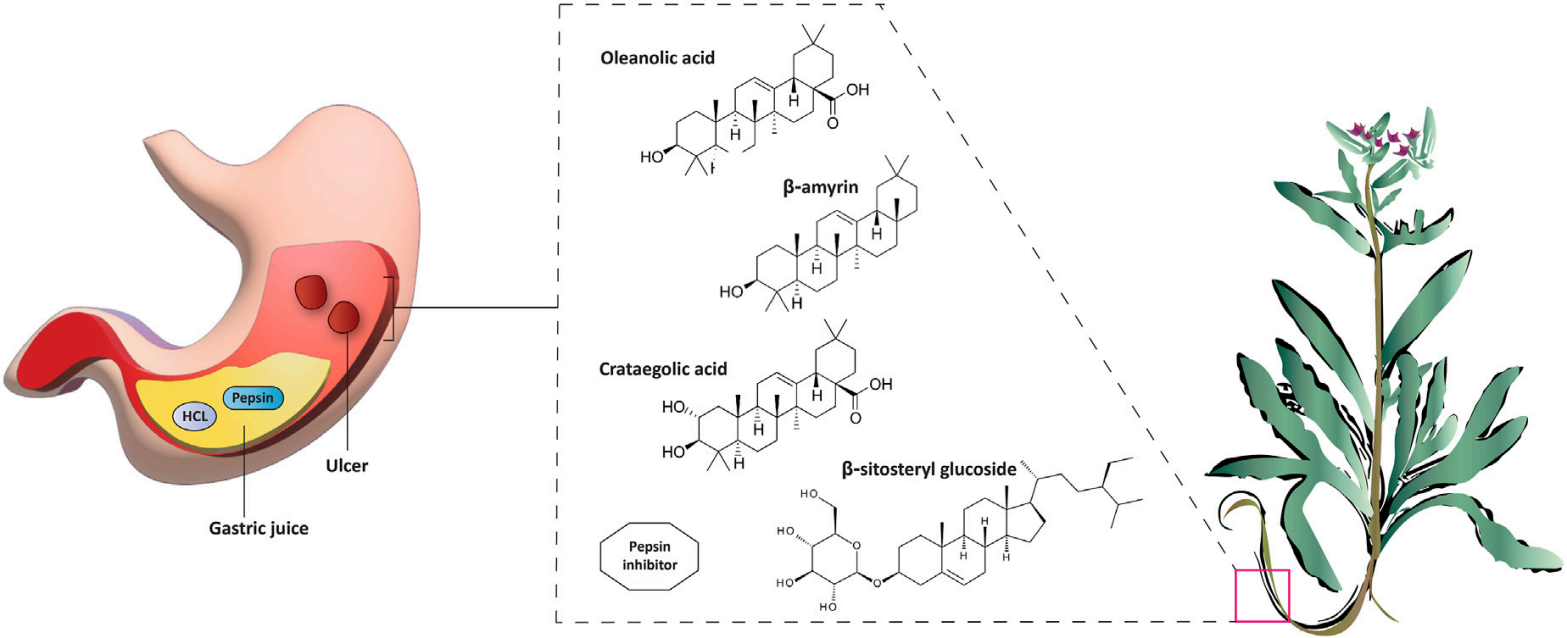
*Anchusa strigosa* has gastroprotective properties. *A. strigosa* root extracts show a protective antigastritis effect by preventing the formation of ulcers in laboratory animals administered with ethanol to cause gastric necrotic damage. The extracts are also effective in the treatment of ulcers. These contain several metabolites that are known to have anti-ulcerogenic effects, including a pepsin inhibitor.

*A. strigosa* has been traditionally used in the treatment of gut and digestive disorders such as abdominal pain, diarrhea, and vomiting. And over the last few decades, several studies have demonstrated its gastroprotective properties, mostly in the management and treatment of gastric ulcers, using particularly the roots (Figure 5; Table 6). For example, in a study using ethanol-induced ulcer in laboratory animals, treatment with an aqueous extract from *A. strigosa* roots before ulcer induction showed a protective antigastritis effect and protected the stomach from ulcer formation, as observed by a lower lesion index (Disi et al., 1998). It was also shown to be effective in the treatment of ulcers, showing even complete healing and recovery within 25 days after ulcer formation (Disi et al., 1998). Fractionation studies were carried out on the *A. strigosa* root extract in an effort to identify the metabolites responsible for its gastroprotective effect. As such, four triterpenoids were isolated from the petroleum ether fraction, which showed strongest activity, and were identified as: oleanolic acid, β-amyrin, crataegolic acid, and β-sitosteryl glucoside (Abbas et al., 2009). These have been previously isolated from various medicinal plants and were shown to have strong anti-ulcerogenic effects (Xiao et al., 1992; Navarrete et al., 2002; Arrieta et al., 2003; Rodríguez, 2003). Furthermore, a pepsin inhibitor was isolated from the aqueous extracts of *A. strigosa* roots, confirming the gastroprotective effect of *A. strigosa* (Abuereish, 1998). In fact, excessive secretion of acid and pepsin is a major cause of hemorrhagic damage of the gastric mucosa and at the root of the genesis and chronicity of ulceration. As such, inhibition of pepsin activity has been a major line in the treatment of ulcers. These results suggest that *A. strigosa* extracts and isolated metabolites should be further exploited as a source of potential therapeutic agents in the treatment of gastric ulcers.

### 5.7 Antidiabetic effect

**FIGURE 6:**
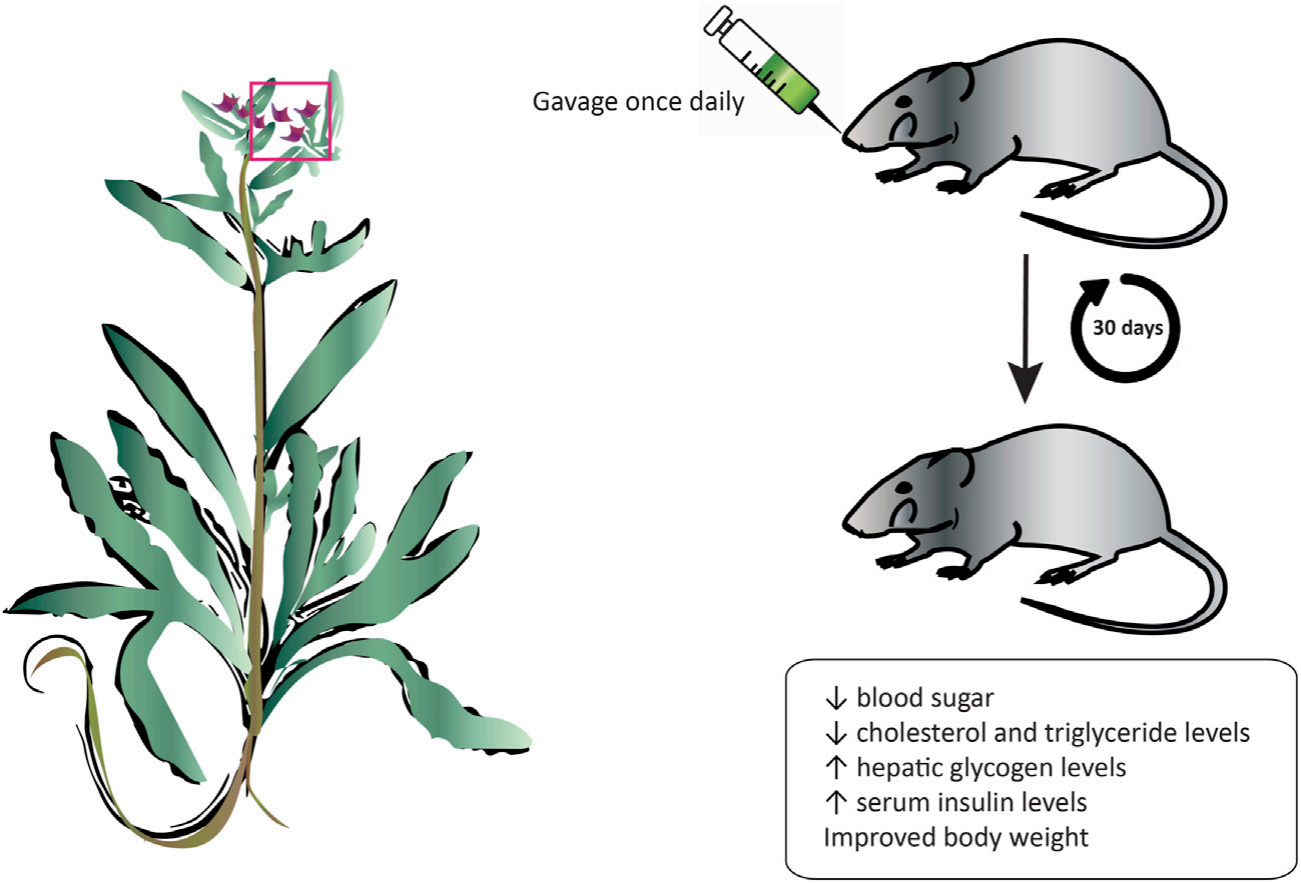
*Anchusa strigosa* exhibits an antidiabetic effect. The oral administration of *A. strigosa* flower extract significantly improved the glycemic and lipidemic profiles of streptozotocin-induced diabetic rats.

Diabetes is a serious metabolic disorder that is caused by a deregulation of blood sugars due to the inefficient production of insulin by the pancreas or when the body is unable to respond and use effectively that insulin. If left uncontrolled, the elevated blood glucose concentration, or hyperglycemia, can lead to serious damage to blood vessels and nerves that can lead to permanent vision loss, kidney failure, and cardiovascular complications. Medicinal plants have long played an important role in the management and treatment of diabetes before the discovery of drugs. Today, there has been a resurgence of public interest in therapies from natural sources due to their cost-effectiveness and associated limited side effects. The oral administration of an aqueous extract from *A. strigosa* flowers significantly decreased blood sugar levels, as well as cholesterol and triglyceride levels in a streptozotocin-induced diabetic rat model (Figure 6; Table 6) (Beyatli and Ari, 2012). Moreover, the body weight of rats improved, and the levels of hepatic glycogen increased, possibly due to the reactivation of glycogen synthase and observed increase in serum insulin levels. These results show that *A. strigosa* flowers could be a potential source of anti-hyperglycaemic and hypolipidemic agents in the treatment of diabetes that warrants further investigation.

### 5.8 Other activities

Over the last decade, nanotechnology has been gaining tremendous impetus and holds great promise in various domains, including technology industries, agriculture, food, cosmetics, and medicine. It involves the manipulation of materials into their nanosize, and making use of their unique chemical, physical, mechanical, and optical properties. However, the synthesis and application of nanotechnology is not without its challenges and available techniques are expensive and require a lot of material and energy. Recently, there has been growing interest in the green synthesis of nanoparticles through the use of microorganisms and plants. These provide active biomolecules that facilitate the bioreduction of metal ions to their elemental form, producing nanoparticles in the 1- to 100-nm range (Zafar, 2023). It is a clean, safe, cost-effective, and environmentally friendly approach that does not require the use of toxic chemicals and high energy processes. Moreover, it results in the production of more stable nanoparticles compared to other traditional synthesis methods that can also support mass production (Huston et al., 2021). Contextually, an *A. strigosa* flower extract was used in the green synthesis of copper oxide nanoparticles. The obtained nanoparticles were very stable and effective in the removal of basic safranine dye from aqueous solutions (Khit et al., 2023).

## 6 Safety of *Anchusa strigosa*

Toxicological studies are crucial when developing a botanical drug to ensure its efficacy and safety profile prior to administration to humans, as these can have adverse effects on the body or interfere with other drugs (Thakkar et al., 2020; Hossain et al., 2022). The incorporation of *A. strigosa* in the Levantine cuisine for many years without any reported toxicity provides some assurance for the safety of this plant (Qasem, 2015; Yeşil et al., 2019; Fullilove, 2022; Baydoun et al., 2023). Moreover, acute toxicity studies of the aqueous *A. strigosa* flower extract showed no mortality or toxic reactions to rats administered orally with the extract at 1, 2, and 4 g/kg of body weight during the 72 h treatment period (Beyatli and Ari, 2012). However, the aqueous extract of *A. strigosa* roots used for the gastroprotective studies showed an intraperitoneal lethal dose 50 (LD50) of 0.08 g extract/kg body weight in mice, which is considered high compared to other plants (Disi et al., 1998). The authors argued that the extract dose required for ulcer treatment is much lower than the lethal dose. In fact, subchronic toxicity studies of this extract on rats showed no histological changes when water intake was replaced with 75 ml of the extract at concentrations of 2.865, 3.57, and 4.284 g/l per animal per day for 90 days, except for observed depressive effects on the central nervous system and general weakness at doses higher than 3.57 g/L. Moreover, the ingestion of a therapeutic dose of 0.286 g/day/kg body weight administered to guinea pigs with ethanol-induced ulcer had no toxic effect on the animals during the 24-day study (Disi et al., 1998). While the acute toxicity tests described here provide a preliminary and positive effects with no obvious toxicity, subacute and chronic studies have demonstrated some side effects. Whereas these toxicology studies provide some reassurance regarding the use of *A. strigosa* as a botanical drug, they fail to highlight the fact that this plant has high levels of PAs, which are a concerning risk and safety issue, particularly related to chronic toxicity. The intake of PAs is associated with liver damage, whereas the prolonged exposure to PAs has been linked with genotoxic and carcinogenic effects. Overall, these observations indicate that further toxicology screenings and additional confirmation testing need to be carried out to validate the safe usage of this plant.

## 7 Conclusion and future perspectives

Plant materials have long been used in the treatment and prevention of human diseases. Now more than ever, plants are seen as potential lead metabolites for drug development. As such, research on plant products has been gaining more and more interest in recent years. However, in plant-derived drug discovery, plant metabolites should be optimized for their efficacy and follow a thorough assessment for their toxicity. Phytochemical and pharmacological studies on *A. strigosa* have shown that it is a rich source of bioactive metabolites with a wide range of applications, further supporting its ethnopharmacological uses. However, *A. strigosa* is also rich in PAs, which are toxins produced by the plant as a defense mechanism against insects and herbivores. Toxicokinetics of PAs show that they are readily absorbed via the gastrointestinal tract and distributed to the liver, where they are metabolized to highly reactive pyrroles that cause damage to the liver, kidneys, and lungs. Therefore, the use of plants rich in PAs has been controversial over their associated risks and safety, impeding their clinical application. In parallel, PAs have been drawing greater attention as promising drug leads due their vast pharmacological properties including anti-microbial, anti-inflammatory, and anti-cancer activities, among others. Therefore, assessing the toxicity profile of *A. strigosa* extracts is of vital importance. And strategies to reduce the toxicity of PAs, while maintaining their bioactivity and improving their therapeutic effect is key to move forward to clinical trials and drug development.
